# Ancestral Alleles in the Human Genome Based on Population Sequencing Data

**DOI:** 10.1371/journal.pone.0128186

**Published:** 2015-05-28

**Authors:** Leeyoung Park

**Affiliations:** Natural Science Research Institute, Yonsei University, Seoul, Korea; Plymouth University, UNITED KINGDOM

## Abstract

Ancestral allele information is useful for genetics studies. Previously, the identification of ancestral alleles was primarily based on sequence alignments between species. Alternative ways to identify ancestral alleles were proposed in this study based on population sequencing data. The methods described here utilized the diversity between haplotypes harboring ancestral and newly emerged alleles. Simulations showed that these methods were reliable for identifying ancestral alleles when the variants had not aged too greatly. Application to the human genome sequencing data suggested the role of indels in maintaining the GC content in the human genome. The deletion-to-insertion ratios and GC proportions were correlated depending on the sizes of insertions and deletions in the direction of increasing GC content. There were GC-biased fixations in single base-pair insertions and AT-biased fixations in single base-pair deletions in the results based on the proposed methods. In the current study, GC-biased gene conversions in nucleotide substitutions were very slight or insignificant. In the variants of several quantitative trait loci (QTLs), slight GC-biased gene conversion was observed in nucleotide substitutions. For the QTL indels, insertions were observed more often than deletions, and deletion-biased fixation was observed, providing new insights into the evolution of functional genes.

## Introduction

How genomes evolve is one of the major questions in biology [1, 2], and knowing which allele is ancestral is important for understanding genome evolution. The actual direction of nucleotide substitutions could provide valuable information on the formation of GC isochores, large DNA segments with low variability in their GC content. Ancestral allele information would be crucial for increasing accuracy when estimating allele ages and could provide a better understanding of genomic signatures due to selection pressures. Knowing the ancestral alleles of variants could also offer more specific explanations regarding the formation of linkage disequilibrium patterns in the genome. In addition, ancestral allele information is also potentially helpful for understanding the rise and extinction of disease-causing variants and disease etiology [3–7].

In previous studies, several related species have been compared in order to identify ancestral alleles [8]. Advances in multiple sequence alignments have allowed up to 84.47% coverage of the human genome, enabling the genome-wide identification of ancestral alleles in the 1000 Genomes Project [9–11]. The alignment is based on sequences from each species, which contain variants and private mutations specific to individuals. At some variant or mutation positions, the alignment may lead to misinterpretation of the ancestral allele. Alternative investigations would be particularly useful for validating insertions and deletions (indels). Deletional biases are well-known features of most genomes [12, 13]. Ancestral allele identification using sequence alignments produces more deletions than insertions due to errors, and based on recent developments, deletional bias persists even after corrections favoring deletions are made [14, 15]. Therefore, even with these enhancements in whole-genome alignment methods, the identification of ancestral alleles still requires improved or alternative methods.

Population genetics information can be used to identify ancestral alleles. The use of a simple pairwise metric of haplotype homozygosity recently demonstrated that derived alleles exhibit significantly higher mean homozygosities than ancestral alleles that were identified using multiple sequence alignments of the data from the International HapMap Project [16]. The HapMap data were targeted for frequent polymorphisms to find tagging variants for the genome-wide association studies (GWAS), and statistics based on allele frequency distributions were not appropriate in the study [16]. When a mutation arises, the haplotype containing the mutant allele is initially monomorphic. As the mutant allele increases in frequency, the haplotypes containing the mutant allele begin to harbor variants due to mutation and recombination. The variant diversity in the mutant haplotypes is usually much less than that in the original haplotypes. The comparison of the population mutation parameters of each type of haplotype, with and without the mutated allele, can reveal which allele is ancestral. Based on the diversity information, two novel methods for identifying ancestral alleles were proposed in this study.

These population-based methods were applied to the human genome sequencing data from the 1000 Genomes Project [17, 18]. The identified ancestral alleles were compared with ancestral alleles based on multiple sequence alignments, and the genome-wide properties of the ancestral alleles based on both methods were studied. Recently, genome-wide RNA sequencing studies were conducted using the same cell lines in the 1000 Genomes Project [19, 20], one of which provided quantitative trait loci (QTL) with high confidence [19]. The ancestral alleles of the QTL were examined to look for any differences from the ancestral alleles in whole genome data. In addition, from the catalog of published genome-wide association studies (GWASs), the GWAS variants were examined for any differences compared with other variants.

## Methods

### Ancestral allele identification

For a variant in a population with N individuals, two types of haplotypes exist: a haplotype harboring a newly emerged allele and a haplotype harboring an ancestral allele. After an allele has emerged and survived, the frequency of the haplotype harboring the newly emerged allele may increase in the population over time. Originally, the haplotype containing the newly emerged allele is monomorphic; over time, the haplotype diversity increases due to mutation and recombination. If the variant survives for a sufficiently long time, both haplotypes become indistinguishable in terms of their diversities. Until then, the haplotype harboring the newly emerged allele shows less diversity leading to a smaller population mutation parameter (θ), than the original haplotype. Ancestral alleles can be identified by measuring the diversity of each haplotype and comparing the results. Therefore, the procedure of identifying ancestral alleles is as follows: 1) estimate θ for each type of haplotypes; 2) compare θ estimates; and 3) designate the allele of the haplotype having the highest θ as an ancestral allele.

In the current study, the θ estimates and simulations are based on the Wright-Fisher population model of a fixed population size with a finite site. Therefore, other estimates that disobey these assumptions were excluded in the current study. The easiest method of measuring diversity is to examine the number of polymorphisms in the haplotype. The measurement can be performed for certain base pair ranges that are equal distances from the variant position. Based on the formula presented below [2, 21], the population mutation parameter (4Nμ), theta (θ), is derived for each haplotype, with and without the newly emerged allele. Among the two estimates for a bi-allelic variant, a smaller theta indicates that the allele in the haplotype is newly emerged.

$$\theta_{1}=\text{ log}\left(\text{1-P}\right)\text{ / log}\left(\text{q}\right)$$

where P represents the proportion of polymorphic sites on a haplotype with a specific allele and q represents the smallest non-zero allele frequency of the haplotype sample.

Another method of measuring diversity is to use Wright's theoretical expression [22]. The population mutation parameter (θ) can be derived from population sequencing data as indicated below, which has been previously described [23]. To determine each estimate, the former theta is indicated as theta1 (θ_1_), and the latter theta is indicated as theta2 (θ_2_). Because recombination introduces new variants into the haplotype, the estimates involve the slight influence of recombination. If the range of measurement is as small as possible to estimate theta, however, the influence of recombination could be minimal.

$$\theta_{2}=\;\frac{1}{2}\left(\frac{m\times (1-m)}{v}-1\right)$$

where m represents the mean of the beta distribution of allele frequencies for a type of haplotype with a specific allele and v represents the variance of the beta distribution for a type of haplotype with a specific allele. Because the type of variants on the haplotye does not need to be distinguishable, the beta distribution is fair in the current study. As with θ_1_ for a bi-allelic variant, smaller estimates indicate that the allele in the haplotype is newly emerged. For a multi-allelic variant, the order of allele emergence is determined depending on the theta estimates of each type of haplotypes.

### Simulations

**Fig 1:**
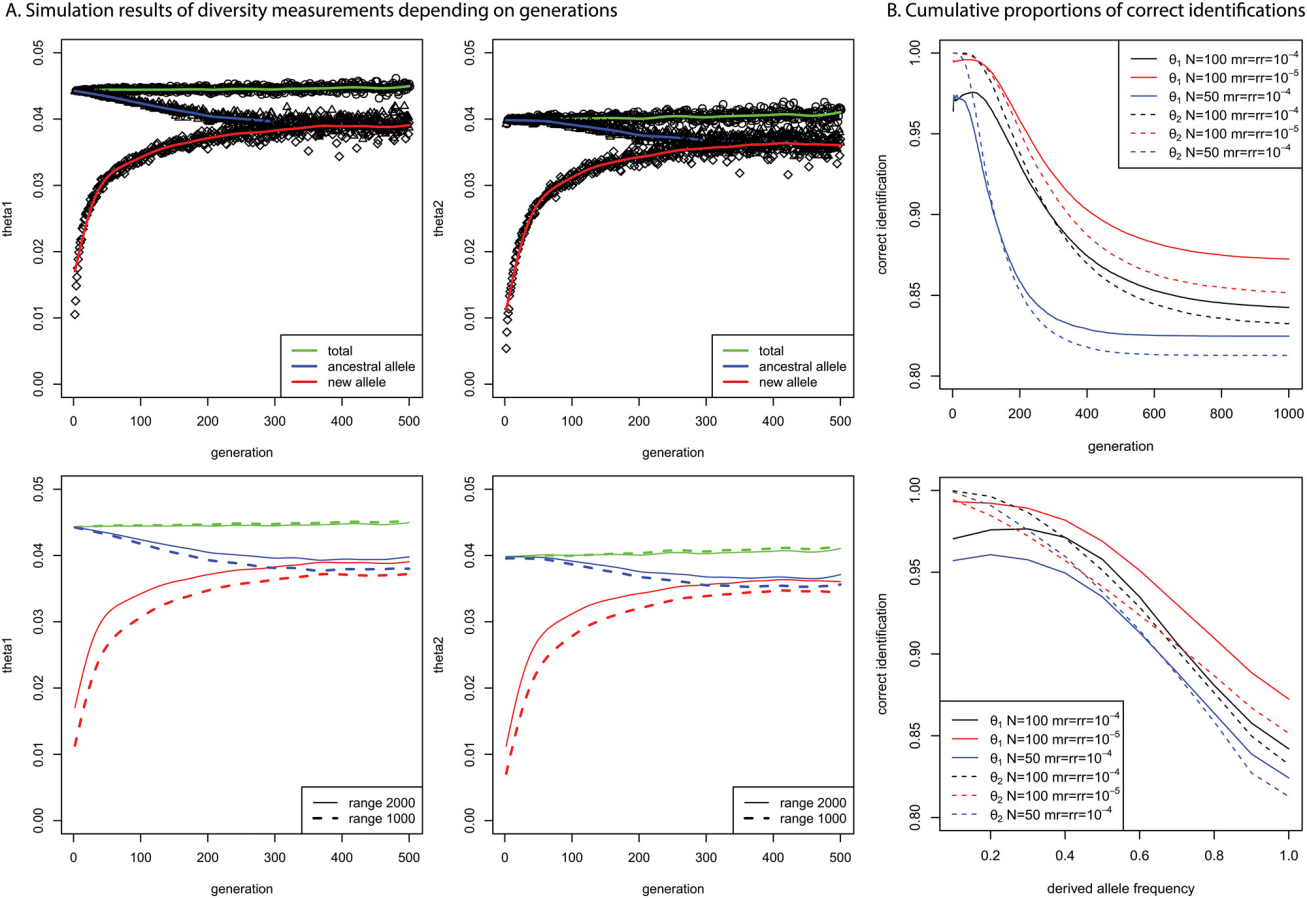
A. Simulation results of diversity measurements for derived and ancestral alleles (N: 100, mutation rate of 1 base pair per generation: 0.0001, recombination rate of 1 base pair per generation: 0.0001); B. Cumulative proportions of correct identification of ancestral alleles according to the generations and derived allele frequencies.

Simulations were performed to examine the validity of the proposed methods, similar to a previous study [24] that was based on a theoretical study [25]. The simulations were conducted assuming constant mutation and recombination rates and a constant population size (N). Due to the computing efficiency and availability, population sizes of 100 and 50 were examined, and the mutation and recombination rates were 0.00001, which were much higher than the actual values for the human genome. The total site was 30000 base pairs (bp), and the estimated range was ±2000 bp from the target variant unless otherwise specified. To begin at an equilibrium state, the initial sequence contained variants based on a beta distribution with parameters of 4Nμ, and random mating with constant mutation and recombination rates was performed for 8N generations. After the initial random mating, random mating for 1000 generations was conducted to examine the diversity of derived and ancestral alleles of variants. The mean estimates of variants at each generation (from 2 to 500) following the simulations are illustrated in Fig 1A and S1 Fig.

Because a sequence of 30000 bp did not have sufficient variants when the mutation and recombination rates were 0.0001, 20 sequences of 30000 bp were examined together using parallel computing. When the mutation and recombination rates were 0.00001, 300 sequences of 30000 bp were analyzed together. For the sampling simulations, the same simulations for a 2000 bp sequence were performed up to the initial random mating for 8N generations, and samplings with replacement proceeded for different sample sizes. This procedure was repeated 1000 times in parallel, and the mean values and confidence intervals are plotted in S2 Fig.

### Analyses of the human genome sequencing data

Low-coverage whole-genome sequencing data of 1092 individuals produced by the 1000 Genomes Project were used for the analyses [17, 18]. The integrated files of sequencing data of the 1000 Genomes Project contain the value of 0 or 1 as alleles with the information of reference and alternative bases. The frequencies (f) of variants with the target allele are ordered and evenly assigned to either f or 1-f for a fair beta distribution. Considering the low mutation and recombination rates in the human genome, the ranges around the target variants were set to ±5,000 bp for both θ_1_ and θ_2_ estimates. The estimates required a sufficient number of variants within the range. If the region near the target was not sufficiently covered (more than 3,000 bp missing), the target variant was not included in the estimations. From the sampling simulations, variants with both alleles having more than 10 allele counts (minor allele frequency>0.0046) were included in the θ_1_ estimates, and variants with both alleles having more than 20 allele counts (minor allele frequency>0.0092) were included in the θ_2_ estimates.

The data from the 1000 Genomes Project contain the ancestral allele information based on multiple sequence alignments [9–11]. The ancestral sequences were inferred based on the EPO (Enredo, Pecan, Ortheus) pipeline using four primate sequences (i.e., human, chimpanzee, orangutan, and rhesus macaque) [9–11]. The calls of ancestral alleles were based on the sister and the ancestral sequences. For accuracy, only ancestral alleles with high certainty based on sequence alignments were included for analyses, which are indicated with uppercase letters. The high certainty means the agreement of all three sequences, namely, the human-chimpanzee ancestral sequence, the chimpanzee sequence, and the human-chimpanzee-orangutan ancestral sequence (ftp://ftp.1000genomes.ebi.ac.uk/vol1/ftp/pilot_data/technical/reference/ancestral_alignments/README). The analyzed variants are summarized in Table 1.

**Table 1:** Numbers of analyzed variants and mean estimates of total haplotypes, including the derived and ancestral alleles, to identify the ancestral alleles in the human genome.

Chr no	Total variants	Seq-alignment	θ_1_	θ_2_	θ_1_ mean	θ_2_ mean
**chr1**	3007196	2681889	1332369	1033442	0.00186	0.00101
**chr2**	3307592	2977486	1438337	1112386	0.00192	0.00101
**chr3**	2763454	2507316	1215693	945268	0.00195	0.00106
**chr4**	2736765	2446564	1238264	966329	0.00202	0.00116
**chr5**	2530217	2271068	1112596	858517	0.00196	0.00105
**chr6**	2424425	2144340	1109074	872262	0.00213	0.00158
**chr7**	2215231	1921144	1006225	780106	0.00200	0.00113
**chr8**	2183839	1943128	951217	739185	0.00225	0.00120
**chr9**	1652388	1474360	738226	572214	0.00206	0.00111
**chr10**	1882663	1666382	851449	664520	0.00201	0.00113
**chr11**	1894908	1634128	843353	654685	0.00200	0.00110
**chr12**	1828006	1608863	823997	640900	0.00192	0.00106
**chr13**	1373000	1238608	622262	484815	0.00195	0.00110
**chr14**	1258254	1104445	564434	440042	0.00195	0.00108
**chr15**	1130554	988861	505519	390422	0.00199	0.00109
**chr16**	1210619	1052238	534800	411785	0.00240	0.00130
**chr17**	1046733	892498	467423	363514	0.00189	0.00104
**chr18**	1088820	988151	488144	380519	0.00198	0.00110
**chr19**	816115	551602	393535	310703	0.00204	0.00127
**chr20**	855166	763634	380929	295964	0.00196	0.00107
**chr21**	518965	453443	239236	187792	0.00210	0.00123
**chr22**	494328	398600	235771	184880	0.00207	0.00124
**Sum or Mean**	38219238	33708748	17092853	13290250	0.00202	0.00114

### Analyses of the QTL and GWAS data

A recent study identified functional variants using the RNA sequencing of lymphoblastoid cell lines from the same individuals in the 1000 Genomes Project [19]. Due to the intrinsic statistical properties, nearly all of the QTL had high minor allele frequencies; therefore, most of the QTL variants had ancestral allele information from both the θ_1_ and θ_2_ estimates. The QTLs in which duplicates in their positions were eliminated were used for the data analyses. The number of total variants that were examined is indicated in Table A in S1 File. There were two data sets, EUR and YRI, and the allele frequencies of the corresponding populations in the 1000 Genomes Project were used to interpret the ancestral allele frequencies. The GWAS variants were downloaded from the GWAS catalog (https://www.genome.gov/26525384). The GWAS variants had high minor allele frequencies due to the same intrinsic statistical property as the QTLs. The number of total GWAS variants in which duplicates in their positions were eliminated was 11910, and the total number of analyzed variants was 11833.

## Results

### Ancestral allele identification

The simulation studies confirmed the validity of methods for identifying ancestral alleles by measuring haplotype diversity. Fig 1A shows the simulation results of the two estimates when both the mutation and recombination rates were 0.0001 and the population size was 100. As a variant became old, the diversity of haplotypes containing the derived allele increased, and the estimates finally equaled the estimates of haplotypes containing ancestral alleles. The θ_2_ estimates of all the haplotypes were almost 0.0004, which is the same as 4Nμ, where N is the population size and μ is the mutation rate. However, θ_1_ estimates were usually slightly higher than 4Nμ for various mutation rates and population sizes, which was likely because of the assumption of the infinite number of allelic states when deriving θ_1_ [21].

In Fig 1A, the diversities of both ancestral and derived haplotypes became indistinguishable as the generation reached 300 when the population size was 100, which is shorter than the average time until fixation (4N) [21]. The estimates in Fig 1A were based on the diversity measure, ranging from ±2000 bp around the target variant. The estimates of the total variants or variants at equilibrium were nearly constant, with ranges of either ±1000 bp or ±2000 bp, as shown in Fig 1A. However, the time until convergence between the estimates of derived and ancestral alleles was slightly longer when a smaller range (±1000 bp) was applied. Therefore, a smaller range would produce more accurate identifications of ancestral alleles. The time until convergence became longer as the population size increased or the mutation and recombination rates decreased (S1 Fig). Therefore, a longer period of time would be expected for the convergence in the human genome, meaning increased accuracy in identifying ancestral alleles for larger population sizes, smaller mutation rates, and smaller recombination rates.

Fig 1B shows the proportions of correct identifications of ancestral alleles for the variants with theta estimates. There were few variants having large generations or large derived allele frequencies. Therefore, the proportions were cumulative according to increasing generations and increasing derived allele frequencies, to examine the proportion of correctly identified ancestral alleles. All the cumulative percentages were above 80%; however, the individual proportions of correct identifications approached approximately 0.5 as the generations approached 1000. As the derived allele frequencies approached 1, the individual proportions of correct identifications dropped rapidly below 0.5, because the identifications are based on population mutation parameters, which are proportional to the population size. However, simple corrections based on allele frequencies are not appropriate in the current method because the identification of ancestral alleles naturally depends on the history of the allele counts of the corresponding alleles. In Fig 1B, the slightly lower proportions of correct identifications using θ_1_ estimates for initial generations and small derived allele frequencies were most likely due to the intrinsic bias of θ_1_ estimates for small population (or sample) sizes similarly shown in sampling simulations.

The simulation results using θ_1_ estimates showed higher percentages of correct identifications than the simulation results using θ_2_ estimates. Under the simulation conditions in Fig 1A, ancestral alleles were correctly identified for 84% of total variants aged up to 1000 generations using θ_1_ estimates and for 83% of total variants using θ_2_ estimates. For smaller mutation and recombination rates of 0.00001, ancestral alleles were correctly identified for 87% of total variants using θ_1_ and for 85% of total variants using θ_2_ estimates. Because the mutation and recombination rates are much smaller in the human genome, the percentage of correct identifications would be higher than 87% and 85% for θ_1_ and θ_2_ estimates, respectively. For a smaller population size of 50, the accuracies decrease to 83% and 81% of total variants with θ_1_ and θ_2_ estimates, respectively. Based on the θ_2_ estimates in Fig 1B when the mutation and recombination rates were 0.00001, the cumulative proportions of correct identifications depending on derived allele frequencies were initially smaller and became larger than the proportions when the mutation and recombination rates were 0.0001 as derived allele frequencies increased. This observation indicates that the proportions of correct identifications using θ_2_ estimates with smaller mutation and recombination rates would be substantially high when derived alleles are major alleles.

In simulations, few variants persisted up to 1000 generations. The ages of most variants were less than 200 generations (80% of total variants) when both the mutation and recombination rates were 0.0001 and the population size was 100 (S3 Fig). As the population size became smaller, there were slightly fewer old variants. Conversely, as the mutation and recombination rates decreased, the number of old variants increased slightly. The derived allele frequencies were distributed densely in low frequencies and sparsely in higher frequencies (S3 Fig). As the population size decreased and mutation and recombination rates increased, the proportion of variants with high frequencies of derived alleles increased. If the mutation and recombination rates were sufficiently large with a small population size, the derived allele frequencies would be distributed almost uniformly, from 0 to 1.

### Ancestral allele identification in the human genome

The proposed methods described above were applied to the human genome sequencing data provided by the 1000 Genomes Project. To obtain accurate identifications considering sampling biases, variants with both allele counts higher than 10 (minor allele frequency>0.0046) were considered for θ_1_, and variants with both allele counts higher than 20 (minor allele frequency>0.0092) were considered for θ_2_. Therefore, the coverage was smaller than that of ancestral allele identification using multiple sequence alignments (Table 1). As observed in the simulations (S1 Fig), the mean of θ_1_ of the total (0.00202) was higher than that of θ_2_ (0.00114) (Table 1). However, the difference was much larger than in the simulations, which was most likely a result of differences in the effective population sizes as well as mutation and recombination rates, attributed to the theoretical difference. The θ_1_ and θ_2_ estimates were slightly higher than those of a previous study using the same estimates of human gene loci [23], indicating that fewer mutations might occur in gene regions.

The major advantage of sequence alignments is their applicability to even private polymorphisms. For variants having both allele counts more than 10, ancestral alleles were identified for almost all of the variants through θ_1_ estimates (99.997% of the chromosomal average) compared to identifications through sequence alignments (84.73%). For variants with both allele counts more than 20, ancestral alleles were identified for 99.97% of the variants based on θ_2_ and 83.83% of the variants based on sequence alignments. The concordances between the two different methods were examined for polymorphisms, for which both methods produced ancestral alleles. The concordances were as follows: 0.86 (number of concordant variants: 11454839) for θ_1_ and θ_2_; 0.89 (12869196) for θ_1_ and sequence alignment; and 0.85 (9516153) for θ_2_ and sequence alignment. When all three methods were available, the concordance was 0.79 (number of concordant variants: 8877059). When more than any two methods identified the ancestral allele, the coverage was 97% (number of corresponding variants: 16086070). Therefore, the population genetic methods can complement existing methods when the ancestral allele from the sequence alignment is unavailable or uncertain.

**Fig 2:**
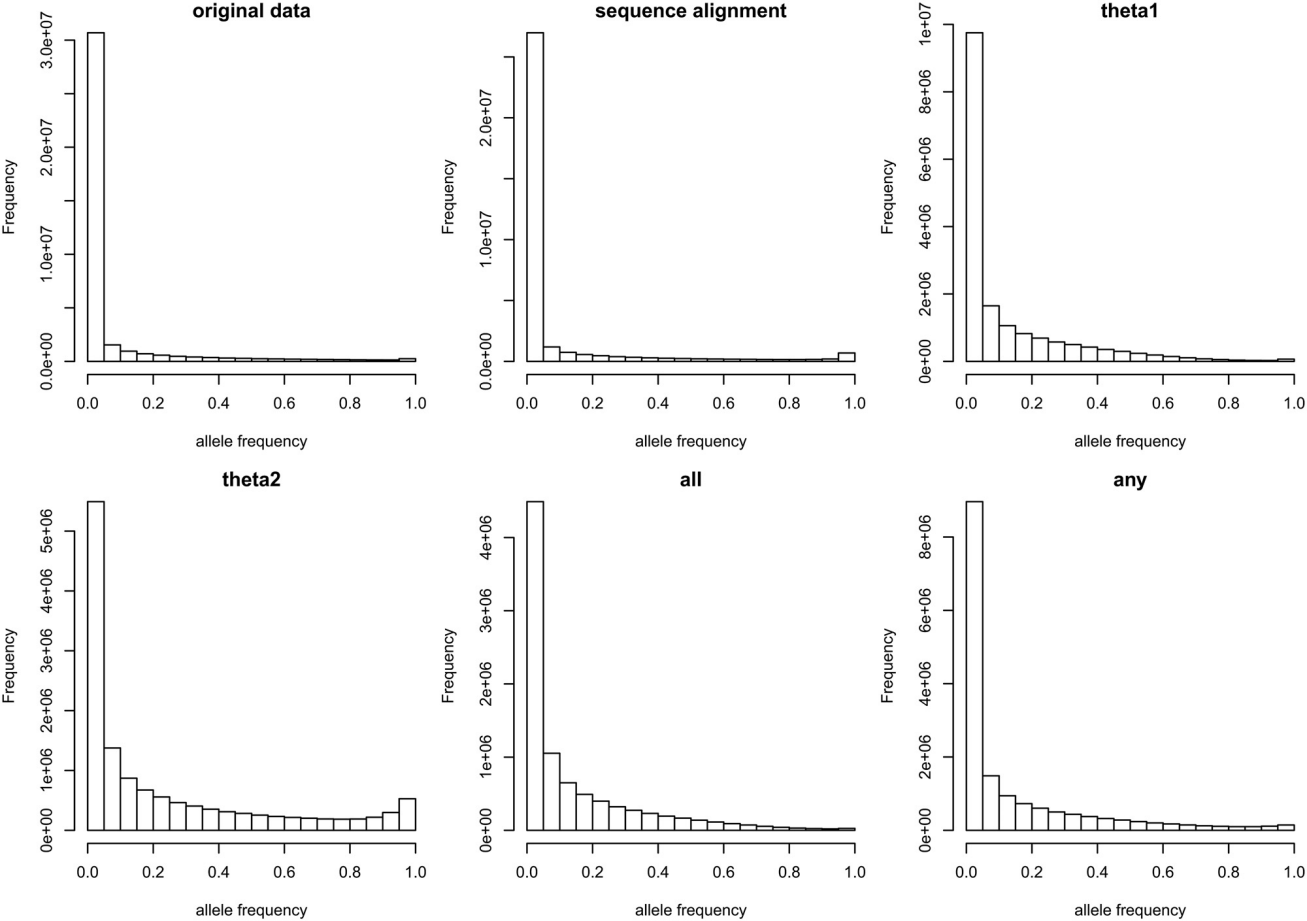
Histograms of derived alleles based on various methods of ancestral allele identification (original data: allele frequency distribution from the sequencing data of the 1000 Genomes Project; sequence-alignment: derived allele frequency distribution by sequence-alignment; θ_1_: the derived allele frequency distribution by θ_1_ estimates; θ_2_: the derived allele frequency distribution by θ_2_ estimates; all: derived allele frequency distribution for the variants with all three methods concordant; any: derived allele frequency distribution for the variants with more than any two methods concordant).

The distributions of derived alleles in Fig 2 were presented to examine the overall accuracy of each method. Because variants are identified in comparison to one reference sequence, it is natural to see a slight bump near 1 in the allele frequency distribution, due to certain homozygotes of rare alleles in the reference sequence. The bump becomes smaller as the sample size increases. Because 1092 individual genomes were sequenced in the 1000 Genomes Project, the bump was very small, as shown in the original data presented in Fig 2. The distribution of newly emerged alleles should not have a bump, similar to the distributions in the simulations (S3 Fig). The derived allele distribution based on the sequence alignment and θ_2_ showed bumps near 1 that should not be seen in the derived allele distributions. The derived allele distribution based on θ_1_ did not have an obvious bump. Considering that the actual mutation and recombination rates in the human genome were much smaller than those in the simulations, the derived allele distribution when all three methods agreed appeared to be approximately correct. The frequency distribution of derived alleles that was at least concordant using more than any two methods showed a slightly more dispersed shape.

The ancestral allele identification made it possible to examine the substitution directions between two bases, and confirmed a clear tendency of increases in A/T compared with G/C in the human genome through nucleotide substitutions (Table 2). Substitutions from G/C to A/T were 58–59% in all transitions (~68%) and 55–56% in all transversions between A/T and G/C. The current methods rely on phased sequencing data. Therefore, the accuracy of phasing is critical for these methods. Although the phase 1 integrated call data from the 1000 Genomes Project were used for the analyses, SHAPEIT2 phased data in the 1000 Genomes Project [26] were tested for comparison. The analyses were performed for chromosome 20 for θ_2_ using the range ±10000 bp. The number of identified ancestral alleles was slightly lower in the results using the SHAPEIT2 phased data: 295620 for θ_2_ in the phase 1 integrated call data versus 269576 in the SHAPEIT2 phased data. The concordance between the two datasets for the calculated variants in both data sets was 0.981.

**Table 2:** Number of nucleotide substitutions for each type in the human genome (Rate: AT mutation rate bias; Expected GC: expected GC proportion from the AT mutation rate bias).

Methods(% Transitions)Rate/Expected GC	Direction	AG	CT	AC	GT	AT	CG
Seq-align	X → Y	4681715	6561124	1178641	1521824	1136407	1417275
(0.682)	X ← Y	6586189	4674730	1531910	1174490	1137087	1412037
1.99/0.33	Total	11267904	11235854	2710551	2696314	2273494	2829312
θ_1_	X → Y	2206576	3162284	567220	708308	536149	674353
(0.684)	X ← Y	3175293	2206561	714588	566844	538419	671601
2.01/0.33	Total	5381869	5368845	1281808	1275152	1074568	1345954
θ_2_	X → Y	1732983	2392367	443313	545528	416808	520360
(0.682)	X ← Y	2399720	1732124	548508	442025	418021	519494
1.95/0.34	Total	4132703	4124491	991821	987553	834829	1039854
All	X → Y	1176686	1684162	305448	386391	289539	367475
(0.680)	X ← Y	1687828	1177402	389332	305098	289983	367192
2.01/0.33	Total	2864514	2861564	694780	691489	579522	734667
Any	X → Y	2088389	3009224	540251	676928	509582	642936
(0.683)	X ← Y	3020729	2088422	682527	539225	512084	641544
2.02/0.33	Total	5109118	5097646	1222778	1216153	1021666	1284480

### Insertions and deletions in the human genome

Deletional biases are well-known features of most genomes [12, 13]. Table 3 provides a summary of the numbers of derived alleles that represented insertions or deletions. Compared with other methods and with the number of identified ancestral alleles in nucleotide substitutions using sequence alignments, the sequence alignment method identified substantially fewer ancestral alleles for insertion/deletion variants. The number of identified ancestral indels in whole chromosomes from the sequence alignments was only half of that from θ_1_ estimates (Table 3). In addition, as shown in Table 3, the sequence alignments identified more deletions than insertions compared to the θ_1_ and θ_2_ estimates.

**Table 3:** Summary of insertions and deletions in the human genome (ratio: deletion/insertion).

Methods	insertion	deletion	ratio	inserted base	deleted base	ratio (base)	% GC insertion	% GC deletion
**Seq-align**	204282	491037	2.40	402554	1221722	3.03	0.349	0.365
**θ**_**1**_	534245	830413	1.55	2217770	23439382	10.57	0.372	0.399
**θ**_**2**_	463693	715306	1.54	3993462	14434339	3.61	0.403	0.398
**All**	125424	325099	2.59	251301	825430	3.28	0.347	0.364
**Any**	408917	725312	1.77	1245483	14295582	11.48	0.366	0.398
**θ**_**1**_ **&θ**_**2**_	352473	596505	1.69	1138427	13981366	12.28	0.369	0.399

The number of deletions was at least 1.5-fold the number of insertions. When the derived alleles that were concordant in all three methods were examined, the number of deletions was 2.59-fold larger than the number of insertions, most likely due to the large deletion-to-insertion ratio from the sequence alignment method (Table 3). Because the numbers of concordant insertions and deletions in all three methods are limited to the numbers of insertions and deletions based on multiple sequence alignments, the derived alleles that were concordant in θ_1_ and θ_2_ were examined as well, and this analysis resulted in a 1.69-fold deletion bias. These deletion biases were much larger than a recent report based on the sequence alignment that could correct errors favoring deletions, in which the deletion biases were only 1.11 for noncoding sequences (ancestral repeats) and 1.29 for coding sequences in the human genome [15].

The total bases in insertions and deletions are listed in Table 3; the number of deleted bases was 3.28-fold larger than the number of inserted bases for the derived alleles that were concordant using all three methods. For the derived alleles concordant using θ_1_ and θ_2_, the number of deleted bases was increased to 12.28-fold the number of inserted bases. The increment seemed to come from several long deletions. The GC content of indels (0.35~0.40) was slightly lower than the GC content of the human genome (0.41) [1], and the GC content was slightly higher in deletions than in insertions for all of the methods except for θ_2_.

**Fig 3:**
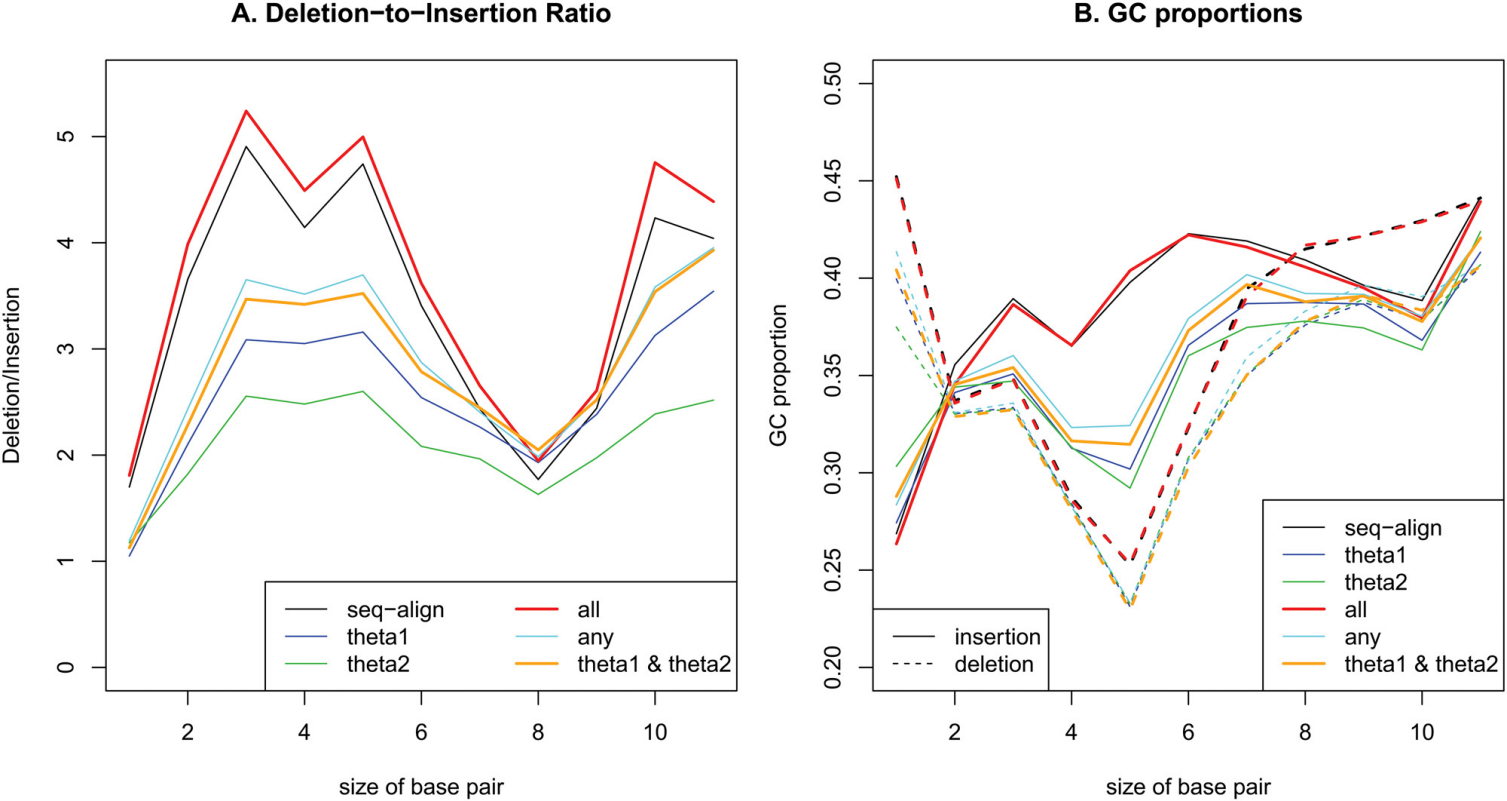
Changes in deletion-to-insertion ratios (A) and GC proportions (B) depending on the size of inserted or deleted base pairs from 1~10 to >10.

When the bp sizes of deleted or inserted sequences were considered, the deletion-to-insertion ratios showed a different pattern depending on the sizes (Fig 3A). The ratios increased for bp sizes of 1 to 3, decreased for bp sizes of 5 to 8, and then increased again. The GC proportion of total insertions and total deletions also changed depending on the bp sizes (Fig 3B). Interestingly, the pattern of insertions was opposite that of deletions. If the GC proportion was high in insertions, then the GC proportion was low in deletions. A more interesting feature was that the pattern of change of the GC proportions corresponded to the pattern of change of the deletion-to-insertion ratio. When the deletion-to-insertion ratio was high, the GC proportion of deletions was lower than that of insertions. These observations indicated the influences of indels in maintaining the GC content in the human genome.

### The biased gene conversion

Previous studies have suggested that GC-biased gene conversion through recombination is the primary explanation for the occurrence of GC isochores in the genome [27–29]. The influence of recombination on nucleotide substitutions strongly supports GC-biased gene conversion [30–32]. However, it has been argued that the fixation bias towards GC is an artifact of parsimony assumptions in sequence alignments between species [33]. To examine GC-biased gene conversion, the changes in AT bias were examined with respect to allele frequencies. The AT bias was calculated as indicated previously [1, 34]. To examine the trend dependent on allele frequencies, the AT bias of substitutions was calculated for each allele frequency range of 0.1. If GC-biased gene conversion exists, the AT bias should decrease as the allele frequency increases.

**Fig 4:**
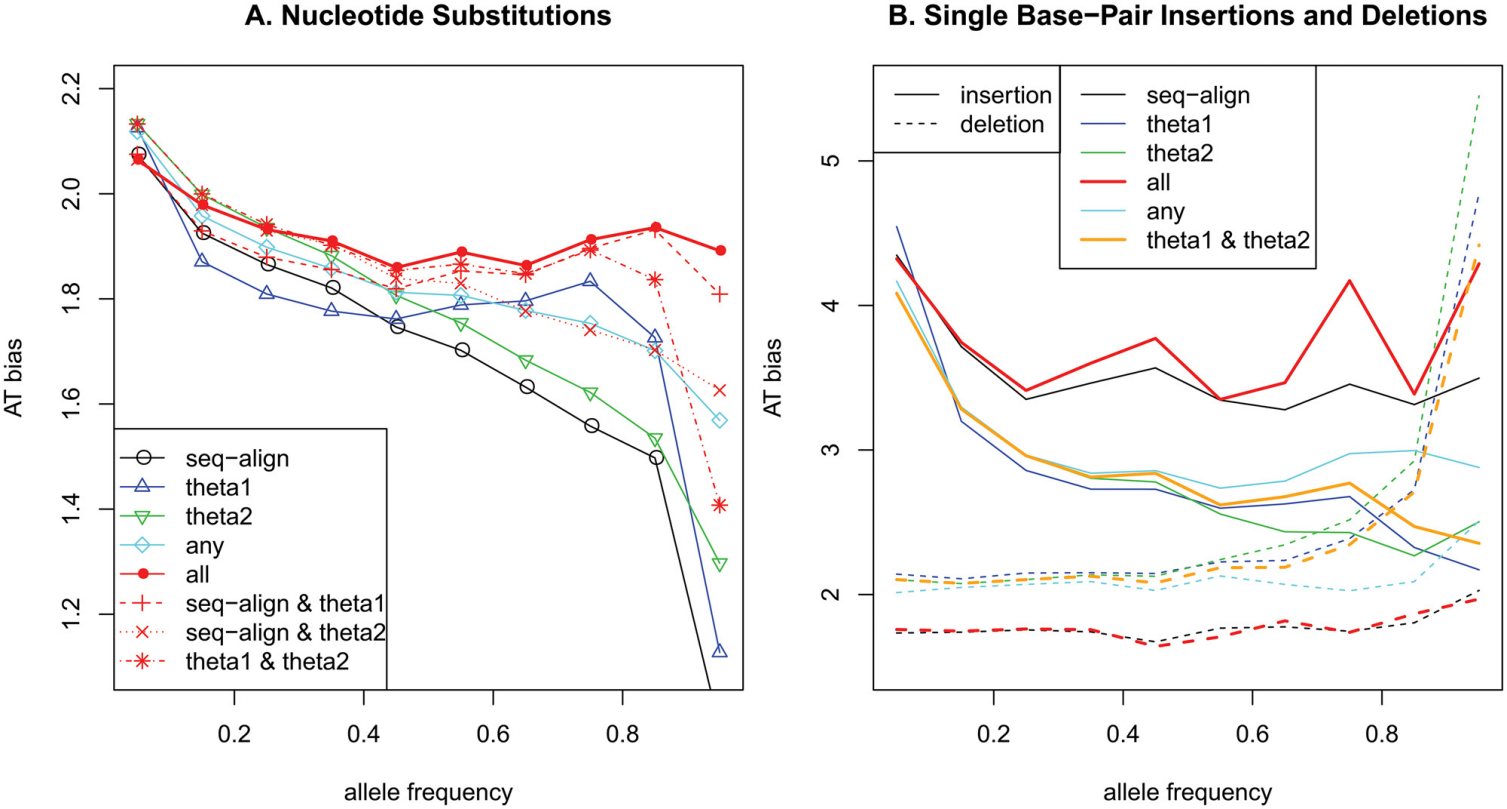
AT bias depending on derived allele frequencies: A. nucleotide substitutions; B. single base-pair insertions and deletions.

In Fig 4, the derived alleles from each method showed GC-biased gene conversion. The derived alleles based on sequence alignments and θ_2_ showed substantial trends in GC-biased gene conversion; whereas the derived alleles based on θ_1_ showed only a slight trend. Notably, derived alleles that were concordant in any two methods always showed lower trend in GC-biased gene conversion. In addition, derived alleles that were concordant in all three methods showed almost no obvious GC-biased gene conversion. The little evidence of biased gene conversion resulted primarily from the derived alleles of θ_1_ estimates. The GC-biased gene conversion of the derived alleles from the sequence alignments was previously argued as an incorrect result [33]. The GC-biased gene conversion of derived alleles from θ_2_ resulted from misidentified ancestral alleles, as shown in Fig 2. The proportion of misidentified alleles increased as the allele frequencies increased in Fig 2, and the actual number of G/C to A/T substitutions would be higher than the actual number of A/T to G/C substitutions among the misidentified derived alleles. Because of the high proportion of misclassified G/C to A/T substitutions, the AT bias in derived alleles decreased as the allele frequencies increased. The reduced decrement of AT bias for derived alleles with high frequencies when both sequence alignment and θ_2_ were consistent supported the condition of very weak GC-biased gene conversion. The absence of GC-biased gene conversion was more apparent in transversions than in transitions (S4 Fig).

In this study, the biased gene conversion in insertions and deletions was also examined. Here, the AT biases of combined insertion bases and deletion bases for single base-pair indels were calculated. Using all indel variants, no clear trend was observed; however, when the variants were grouped depending on their sizes, clear trends were found for single base-pair insertions and deletions. As shown in Fig 4B, the insertions showed the GC-biased gene conversion overall. The result using multiple sequence alignments showed the weakest GC-favoring fixation bias in insertions. Due to the smaller number of indel variants based on sequence alignment, there was a relatively small number of variants with high allele frequencies of the derived alleles when all three methods agreed. In addition, the GC% of one base-pair insertions using the multiple sequence alignments was lowest among the three methods in Fig 3B, which led to high AT biases regardless of the derived allele frequencies. For the small number of variants with high derived allele frequencies based on multiple sequence alignments, the large proportions of A/T insertions provided drastically increased numbers of A/T insertions in variants when all three methods agreed. However, the derived alleles based on both θ_1_ and θ_2_ showed obvious GC-biased gene conversion.

Interestingly, AT-biased gene conversion was observed in the deleted alleles. Again, the result using multiple sequence alignments showed the weakest AT-biased gene conversion. Similar to the one base-pair insertions, it is most likely due to the small number of variants using multiple sequence alignments and the highest GC proportion of one base-pair deletions using multiple sequence alignments among all three methods. The GC-favoring fixation bias in insertions and the AT-favoring fixation bias in deletions indicated the possibility of maintaining the GC contents in the human genome. It could be effective enough to maintain the GC contents, considering that the deletion-to-insertion ratio of single bp indels was the lowest and that the GC contents of single bp indels was high in insertions and low in deletions, as shown in Fig 3. These effects disappeared for indels of more than 2 bp.

### Quantitative trait loci (QTL) and genome-wide association variants

**Fig 5:**
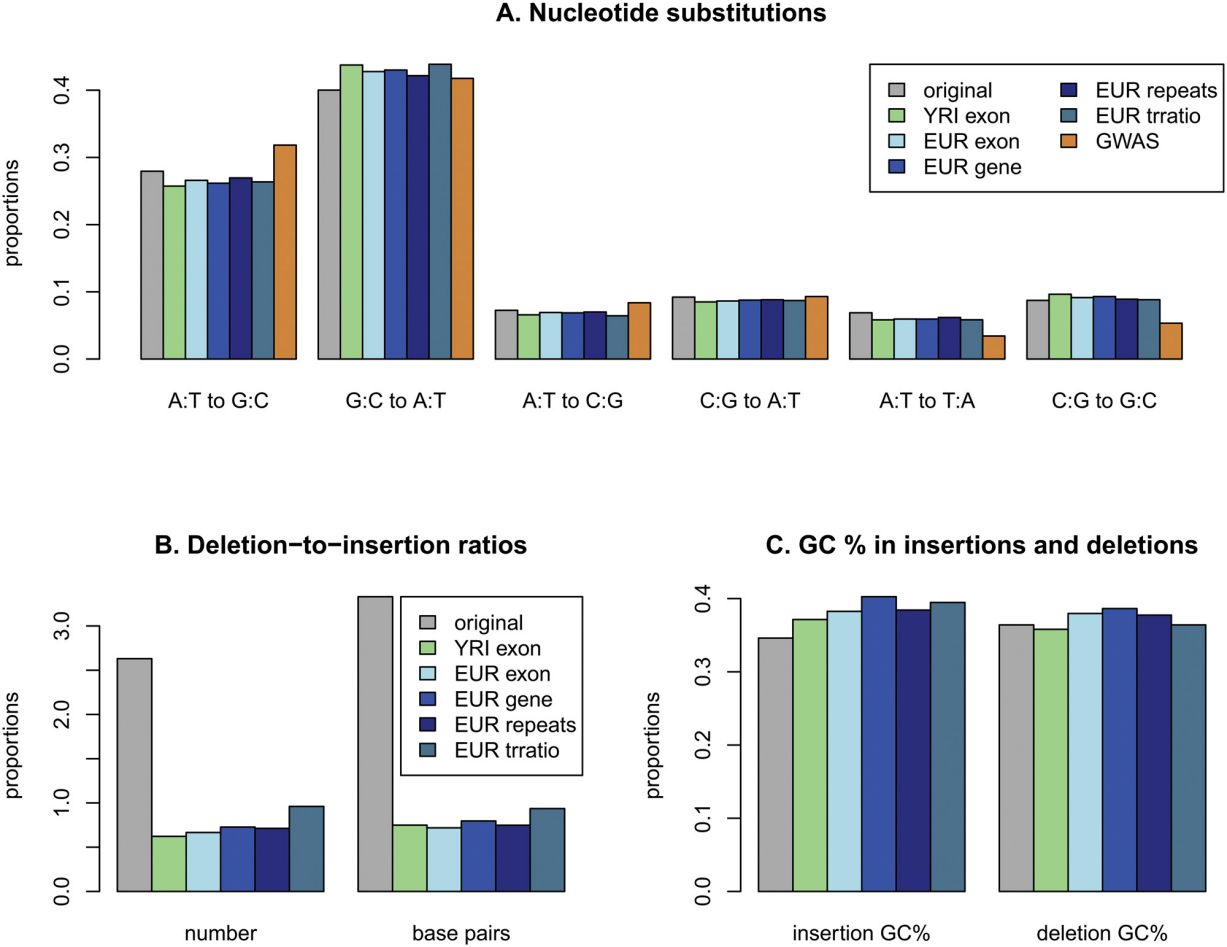
Summary of derived alleles from QTL and GWAS variants: A. Types of nucleotide substitutions; B. Deletion-to-insertion ratios of QTL variants; C. GC proportions in insertions and deletions of QTL variants (original: whole genome data; EUR exon: EUR exon-expression QTL data; YRI exon: YRI exon-expression QTL data; EUR gene: EUR gene-expression QTL data; EUR repeats: EUR transcribed repeats QTL data; EUR trratio: EUR transcription ratio QTL data; GWAS: GWAS catalog data).

The variants from quantitative trait loci (QTL) and genome-wide association studies (GWASs) were examined for the variants that were concordant in all three methods. The types of substitutions were examined for the QTL and GWAS data. Because the analysis required a sufficient number of variants, QTL data with more than 50000 variants were analyzed, which included all of the QTLs of the EUR data except for the miRNA QTLs and exon QTLs of YRI data (Table A in S1 File). The results using variants that were concordant in all three methods are illustrated in Fig 5 (Table B in S1 File). All of the QTL data showed similar results. The increased G:C-to-A:T transitions in the QTL data might be derived from the high transitions in the CpG sites of gene regions. The higher GC contents in gene regions increased the substitutions from C or G (Fig 5A). The QTL variants showed slight GC-biased gene conversions in several QTLs, especially in repeats QTL of EUR (S5 Fig).

The GWAS data showed more dramatic differences in nucleotide substitutions than the QTL data. Transitions were increased overall and showed more increased A:T-to-G:C transitions than G:C-to-A:T transitions, which might have resulted from the low GC content in the region where GWAS variants were located. Transversions between A/T and G/C were increased overall, and the A:T-to-C:G transversions also increased; however, the proportion was still slightly lower than the C:G-to-A:T transversions. In the GWAS data, the proportions of both the C:G-to-G:C and A:T to T:A transversions decreased.

The indels in the QTL data were analyzed and compared with the indels in the whole genome data (Fig 5B and 5C). Interestingly, insertions occurred more often than deletions in the QTL data. The results showed more severe insertion biases than did the results of a previous study on human-specific indels in coding exons [35]. In addition, the deleted bases were smaller than the inserted bases. In the QTL variants, there was a fixation bias of deletions (S6 Fig). Because the whole genome variants did not show any trends, the functional deletion variants might be under selective pressure. The insertions in the QTL data showed a higher GC content than those in the whole genome data. In contrast to the indels in the whole genome data, the GC content in the deletions was slightly lower than that in the insertions in all of the analyzed QTL data (Fig 5C).

## Discussion

In the current study, ancestral allele identification methods based on population mutation parameters using population sequencing data were developed for actual applications for the first time. Unlike in a previous effort [16], the ability to identify ancestral alleles was studied in earnest in the current study. These methods showed evidence of being effective and especially useful for insertions and deletions. As shown in Fig 2, the distribution of derived allele frequencies showed an approximately natural shape when all three methods agreed. The distributions based on sequence alignment and θ_2_ indicated that the ancestral alleles from these methods could be misclassified. In the diversity estimates, the number of corresponding haplotypes in the population is considered to be the population size, and the theta estimates involve fluctuations in allele frequencies in the past. Therefore, the haplotypes with minor alleles could have less diversity than the haplotypes with major alleles. θ_1_ estimates account for the number of the corresponding haplotypes by applying the smallest non-zero allele frequency of the sampled haplotypes with interested alleles to the equation; however, θ_2_ estimates do not, resulting in misclassified ancestral alleles.

The estimates of population mutation parameters in the current study were derived based on the Wright-Fisher population model of a fixed population size with a finite site [21, 22]. Other similar estimates that disobey this basic model should be examined for the availability to identify ancestral alleles using simulations based on a certain proper model. A constant population size was assumed; however, in reality, the population size is not constant. In addition, selection pressures were not accounted for in the basic model. For regions under strong selective pressure or for populations undergoing rapid expansion, the detection of ancestral alleles might be less accurate. It should be noted that, theoretically, the proportions of correct identifications cannot be less than 50% no matter how rapid the population sizes increase because the population expansion applies to both alleles; however, as shown in the high-frequency derived alleles, the proportions of correct identifications could be less than 50% for regions under extremely strong positive selection. The selective pressures should be strong enough to increase the derived allele frequencies of most variants in the region much higher than 0.5, which is unlikely in most natural conditions. The influence of changes in population sizes, population structure, and selection pressures should be studied further to examine how accuracy is affected by the conditions. Some of the variants are expected to be too old to be distinguished based on population genetic methods. If an allele age that either separately or concurrently accounts for the influence of selection pressure and demographic changes can be obtained, the accuracy of the identified ancestral alleles can be determined and can be improved.

Compared to the ancestral allele identification obtained using sequence alignment methods, the proposed methods can identify ancestral alleles for any common variants from population sequencing data. The θ_2_ estimates increased consistently as the sample size increased (S2 Fig). By correcting the sampling biases, the estimate would be more useful for increasing the applicability to rare variants. The sampling bias of θ_1_ estimates decreased as the population size increased (S2 Fig). Considering that the recent changes in effective population sizes of human populations were large [24], the ancestral alleles having smaller allele counts could be correctly identified using θ_1_ estimates for young variants. The examined ranges around the target variants were ±5000 bp; however, the range could be reduced to obtain more accurate results depending on the local mutation rates. For the gene regions in particular, mutation rates appear low due to purifying selection, and the accuracy of identifying ancestral alleles could differ. In addition, there are very large insertions and deletions in the human genome. Variants near or in those insertions and deletions could be affected in their population mutation parameters, and adjusting the effects would lead to a more accurate identification of ancestral alleles for large insertions and deletions.

The evidence of GC-biased gene conversion was unclear in the current study. Considering the correlations between recombination and GC-biased gene conversion, additional studies that include the effect of recombination rates would be required to determine the influence of GC-biased conversion. Slight trends of GC-biased gene conversions were shown in variants of several QTLs (S5 Fig). GC-biased gene conversion could result from either selection or recombination [36]. Additional studies that include local recombination rates would provide accurate explanations of the GC-biased gene conversions of functional variants. As previously suggested [30, 37], studies that consider local differences in substitution rates could also provide more accurate information on genome evolution.

The differences in the proportions of substitution types in the exon QTL variants came from the high GC content in gene regions. The majority of the GWAS variants were located in intergenic regions, which might have relatively low GC contents compared to gene regions. However, the low GC content might not fully explain the differences from the whole genome variants, such as increased transitions and decreased proportions of transversions between A and T and between C and G. The different proportions of nucleotide substitutions in the GWAS variants also need careful examination to exclude any artifacts resulting from the data generation processes. Different from the QTL data based on RNA-sequencing, the variants for genome-wide genotyping were selected depending on specific selection criteria, which might result in a biased result.

In contrast to the nucleotide substitutions, the differences in insertions and deletions of the QTL data (i.e., more insertions than deletions, lower GC content in deletions than insertions, and fixation biases of the deletions) are noteworthy. There was a report regarding the fixation bias of insertions in the gene region of *Drosophila melanogaster* [38]. In this previous study, the number of insertions was not higher than the number of deletions in the gene region, but there were more high-frequency insertions than high-frequency deletions. The QTL indels are likely functional among the indels in gene regions, and a recent study on the human genome showed that functional indels are influenced by strong purifying selection pressures [39]. The different results may indicate the differences between functional indels and general indels in gene regions. The strong deletion-biased fixation in the QTL variants indicated that functional deletions might be under stronger selection pressure than functional insertions. Another recent study on the gene regions of *Drosophila melanogaster* indicated that the deletions, but not the insertions, between 11 bp and 30 bp in size tended to be fixed more frequently than did the synonymous mutations [40]. Most insertions and deletions of the QTL variants were less than 5 bp in the current study. Therefore, a comprehensive analysis on the indels in gene regions could provide more solid conclusions.

## Supporting Information

S1 Fig Simulation results of diversity measurements for derived and ancestral alleles: **A**. N: 100, mutation rate: 0.0001, recombination rate: 0.0001, range: 1000; **B**. N:50, mutation rate: 0.0001, recombination rate: 0.0001; **C**. N:100, mutation rate: 0.00001, recombination rate: 0.00001.
(PDF)

S2 Fig Mean estimates and confidence intervals depending on sample sizes for the population sizes of 100 and 500 (horizontal line: the original estimate). (PDF)

S3 Fig Histograms of derived alleles and distributions of allele ages for various parameters: **A**. N: 100, mutation rate: 0.0001, recombination rate: 0.0001; **B**. N:100, mutation rate: 0.00001, recombination rate: 0.00001; **C**. N:50, mutation rate: 0.0001, recombination rate: 0.0001.
(PDF)

S4 Fig AT bias of nucleotide substitutions depending on derived allele frequencies: A. Transitions; B. Transversions.
(PDF)

S5 Fig Examining biased gene conversions of QTL variants: changes in AT bias depending on the allele frequencies of QTL variants. (PDF)

S6 Fig Fixation bias of deletions using the QTL data: changes in deletion-to-insertion ratios depending on allele frequencies. (PDF)

S1 File Supplementary Tables. **Table A.** Number of total variants that were examined (QTL: variants, of which positions were not duplicated in QTL data; Analyzed: variants that were successfully merged with data with ancestral allele information; mi: microRNA; exon: exon expression QTL; gene: gene expression QTL; repeats: gene repeats QTL; trratio: transcription ratio QTL; combined total: all QTLs). **Table B.** Number of nucleotide substitutions for each type of variant when all three methods agreed (DI: deletion and insertion; QTL EUR exon: EUR exon-expresion QTL data; QTL YRI exon: YRI exon-expression QTL data; QTL EUR gene: EUR gene-expression QTL data; QTL EUR repeats: EUR transcribed repeats QTL data; QTL EUR trratio: EUR transcription ratio QTL data; GWAS: GWAS catalog data).
(DOC)

## References

[1] Lynch M. The origins of genome architecture: Sinauer Associates; 2007.

[2] Hartl DL, Clark AG. Principles of Population Genetics. 4th ed Sunderland: Sinauer Associates, Inc.; 2007.

[3] Martinez-Cadenas C, Lopez S, Ribas G, Flores C, Garcia O, Sevilla A, et al Simultaneous purifying selection on the ancestral MC1R allele and positive selection on the melanoma-risk allele V60L in south Europeans. Mol Biol Evol. 2013;30(12):2654–65. Epub 2013/09/21. 10.1093/molbev/mst158 mst158 [pii]. . 24045876

[4] Wang SS, Lu Y, Rothman N, Abdou AM, Cerhan JR, De Roos A, et al Variation in effects of non-Hodgkin lymphoma risk factors according to the human leukocyte antigen (HLA)-DRB1*01:01 allele and ancestral haplotype 8.1. PLoS One. 2011;6(11):e26949 Epub 2011/11/19. 10.1371/journal.pone.0026949 PONE-D-11-12868 [pii]. 22096508 PMC3212525

[5] Lin MW, Lee DD, Liu TT, Lin YF, Chen SY, Huang CC, et al Novel IL31RA gene mutation and ancestral OSMR mutant allele in familial primary cutaneous amyloidosis. Eur J Hum Genet. 2010;18(1):26–32. Epub 2009/08/20. 10.1038/ejhg.2009.135 ejhg2009135 [pii]. 19690585 PMC2987153

[6] Maruta Y, Okayama N, Hiura M, Suehiro Y, Hirai H, Hinoda Y. Determination of ancestral allele for possible human cancer-associated polymorphisms. Cancer Genet Cytogenet. 2008;180(1):24–9. Epub 2007/12/11. S0165-4608(07)00590-0 [pii] 10.1016/j.cancergencyto.2007.09.011 . 18068529

[7] Mahley RW, Rall SC Jr. Is epsilon4 the ancestral human apoE allele? Neurobiol Aging. 1999;20(4):429–30. Epub 1999/12/22. S0197458099000810 [pii]. . 10604434 10.1016/s0197-4580(99)00081-0

[8] Nei M, Kumar S. Molecular evolution and phylogenetics. Oxford; New York: Oxford University Press; 2000 xiv, 333 p. p.

[9] Paten B, Herrero J, Beal K, Fitzgerald S, Birney E. Enredo and Pecan: genome-wide mammalian consistency-based multiple alignment with paralogs. Genome research. 2008;18(11):1814–28. 10.1101/gr.076554.108 18849524 PMC2577869

[10] Paten B, Herrero J, Fitzgerald S, Beal K, Flicek P, Holmes I, et al Genome-wide nucleotide-level mammalian ancestor reconstruction. Genome Res. 2008;18(11):1829–43. Epub 2008/10/14. 10.1101/gr.076521.108 gr.076521.108 [pii]. 18849525 PMC2577868

[11] Danecek P, Auton A, Abecasis G, Albers CA, Banks E, DePristo MA, et al The variant call format and VCFtools. Bioinformatics. 2011;27(15):2156–8. Epub 2011/06/10. 10.1093/bioinformatics/btr330 btr330 [pii]. 21653522 PMC3137218

[12] Gregory TR. Insertion-deletion biases and the evolution of genome size. Gene. 2004;324:15–34. Epub 2003/12/25. S0378111903009570 [pii]. . 14693368 10.1016/j.gene.2003.09.030

[13] Kuo CH, Ochman H. Deletional bias across the three domains of life. Genome Biol Evol. 2009;1:145–52. Epub 2009/01/01. 10.1093/gbe/evp016 20333185 PMC2817411

[14] Loytynoja A, Goldman N. Phylogeny-aware gap placement prevents errors in sequence alignment and evolutionary analysis. Science. 2008;320(5883):1632–5. Epub 2008/06/21. 10.1126/science.1158395 320/5883/1632 [pii]. . 18566285

[15] Laurie S, Toll-Riera M, Rado-Trilla N, Alba MM. Sequence shortening in the rodent ancestor. Genome Res. 2012;22(3):478–85. Epub 2011/12/01. 10.1101/gr.121897.111 gr.121897.111 [pii]. 22128134 PMC3290783

[16] Fry AE, Trafford CJ, Kimber MA, Chan MS, Rockett KA, Kwiatkowski DP. Haplotype homozygosity and derived alleles in the human genome. Am J Hum Genet. 2006;78(6):1053–9. Epub 2006/05/11. S0002-9297(07)63926-3 [pii] 10.1086/504160 16685655 PMC1474085

[17] Abecasis GR, Altshuler D, Auton A, Brooks LD, Durbin RM, Gibbs RA, et al A map of human genome variation from population-scale sequencing. Nature. 2010;467(7319):1061–73. Epub 2010/10/29. 10.1038/nature09534 nature09534 [pii]. 20981092 PMC3042601

[18] Abecasis GR, Auton A, Brooks LD, DePristo MA, Durbin RM, Handsaker RE, et al An integrated map of genetic variation from 1,092 human genomes. Nature. 2012;491(7422):56–65. Epub 2012/11/07. 10.1038/nature11632 nature11632 [pii]. 23128226 PMC3498066

[19] Lappalainen T, Sammeth M, Friedlander MR, t Hoen PA, Monlong J, Rivas MA, et al Transcriptome and genome sequencing uncovers functional variation in humans. Nature. 2013;501(7468):506–11. Epub 2013/09/17. 10.1038/nature12531 nature12531 [pii]. . 24037378 PMC3918453

[20] Pickrell JK, Marioni JC, Pai AA, Degner JF, Engelhardt BE, Nkadori E, et al Understanding mechanisms underlying human gene expression variation with RNA sequencing. Nature. 2010;464(7289):768–72. Epub 2010/03/12. 10.1038/nature08872 nature08872 [pii]. 20220758 PMC3089435

[21] Kimura M, Ohta T. Theoretical Aspects of Population Genetics. Princeton: Princeton University Press; 1971. 5162676

[22] Wright S. Evolution in Mendelian Populations. Genetics. 1931;16(2):97–159. 17246615 10.1093/genetics/16.2.97 PMC1201091

[23] Park L. Relative mutation rates of each nucleotide for another estimated from allele frequency spectra at human gene loci. Genet Res (Camb). 2009;91(4):293–303. Epub 2009/07/31. 10.1017/S0016672309990164 S0016672309990164 [pii]. . 19640324

[24] Park L. Linkage disequilibrium decay and past population history in the human genome. PLoS One. 2012;7(10):e46603 Epub 2012/10/12. 10.1371/journal.pone.0046603 PONE-D-12-11249 [pii]. 23056365 PMC3462787

[25] Weir BS, Hill WG. Effect of mating structure on variation in linkage disequilibrium. Genetics. 1980;95(2):477–88. Epub 1980/06/01. 7203003 10.1093/genetics/95.2.477 PMC1214241

[26] Delaneau O, Howie B, Cox AJ, Zagury JF, Marchini J. Haplotype estimation using sequencing reads. Am J Hum Genet. 2013;93(4):687–96. Epub 2013/10/08. 10.1016/j.ajhg.2013.09.002 S0002-9297(13)00415-1 [pii]. 24094745 PMC3791270

[27] Duret L, Galtier N. Biased gene conversion and the evolution of mammalian genomic landscapes. Annu Rev Genomics Hum Genet. 2009;10:285–311. Epub 2009/07/28. 10.1146/annurev-genom-082908-150001 . 19630562

[28] Eyre-Walker A, Hurst LD. The evolution of isochores. Nat Rev Genet. 2001;2(7):549–55. Epub 2001/07/04. 10.1038/35080577 35080577 [pii]. . 11433361

[29] Galtier N, Piganeau G, Mouchiroud D, Duret L. GC-content evolution in mammalian genomes: the biased gene conversion hypothesis. Genetics. 2001;159(2):907–11. Epub 2001/11/06. 11693127 10.1093/genetics/159.2.907 PMC1461818

[30] Arndt PF, Hwa T, Petrov DA. Substantial regional variation in substitution rates in the human genome: importance of GC content, gene density, and telomere-specific effects. J Mol Evol. 2005;60(6):748–63. Epub 2005/06/17. 10.1007/s00239-004-0222-5 . 15959677

[31] Duret L, Arndt PF. The impact of recombination on nucleotide substitutions in the human genome. PLoS Genet. 2008;4(5):e1000071 Epub 2008/05/10. 10.1371/journal.pgen.1000071 18464896 PMC2346554

[32] Tyekucheva S, Makova KD, Karro JE, Hardison RC, Miller W, Chiaromonte F. Human-macaque comparisons illuminate variation in neutral substitution rates. Genome Biol. 2008;9(4):R76 Epub 2008/05/02. 10.1186/gb-2008-9-4-r76 gb-2008-9-4-r76 [pii]. 18447906 PMC2643947

[33] Hernandez RD, Williamson SH, Zhu L, Bustamante CD. Context-dependent mutation rates may cause spurious signatures of a fixation bias favoring higher GC-content in humans. Mol Biol Evol. 2007;24(10):2196–202. Epub 2007/07/28. msm149 [pii] 10.1093/molbev/msm149 . 17656634

[34] Weller AM, Rodelsperger C, Eberhardt G, Molnar RI, Sommer RJ. Opposing Forces of A/T-Biased Mutations and G/C-Biased Gene Conversions Shape the Genome of the Nematode Pristionchus pacificus. Genetics. 2014 Epub 2014/01/15. genetics.113.159863 [pii] 10.1534/genetics.113.159863 . 24414549 PMC3982703

[35] Chen FC, Chen CJ, Li WH, Chuang TJ. Human-specific insertions and deletions inferred from mammalian genome sequences. Genome Res. 2007;17(1):16–22. Epub 2006/11/11. gr.5429606 [pii] 10.1101/gr.5429606 17095709 PMC1716262

[36] Berglund J, Pollard KS, Webster MT. Hotspots of biased nucleotide substitutions in human genes. PLoS Biol. 2009;7(1):e26 Epub 2009/01/30. 10.1371/journal.pbio.1000026 08-PLBI-RA-2435 [pii]. 19175294 PMC2631073

[37] Karro JE, Peifer M, Hardison RC, Kollmann M, von Grunberg HH. Exponential decay of GC content detected by strand-symmetric substitution rates influences the evolution of isochore structure. Mol Biol Evol. 2008;25(2):362–74. Epub 2007/11/29. msm261 [pii] 10.1093/molbev/msm261 . 18042807

[38] Leushkin EV, Bazykin GA, Kondrashov AS. Strong mutational bias toward deletions in the Drosophila melanogaster genome is compensated by selection. Genome Biol Evol. 2013;5(3):514–24. Epub 2013/02/12. 10.1093/gbe/evt021 evt021 [pii]. 23395983 PMC3622295

[39] Montgomery SB, Goode DL, Kvikstad E, Albers CA, Zhang ZD, Mu XJ, et al The origin, evolution, and functional impact of short insertion-deletion variants identified in 179 human genomes. Genome Res. 2013;23(5):749–61. Epub 2013/03/13. 10.1101/gr.148718.112 gr.148718.112 [pii]. 23478400 PMC3638132

[40] Chong Z, Zhai W, Li C, Gao M, Gong Q, Ruan J, et al The evolution of small insertions and deletions in the coding genes of Drosophila melanogaster. Mol Biol Evol. 2013;30(12):2699–708. Epub 2013/10/01. 10.1093/molbev/mst167mst167 [pii]. . 24077769

