# Supplementary figures and images for: Ancestral Alleles in the Human Genome Based on Population Sequencing Data
Source: PLoS One. 2015 May 28;10(5):e0128186. doi: 10.1371/journal.pone.0128186 (PMC4447449; doi:10.1371/journal.pone.0128186)

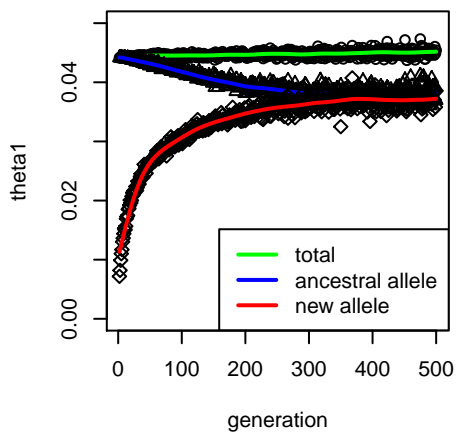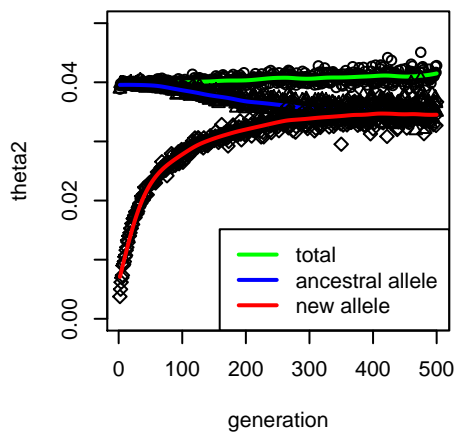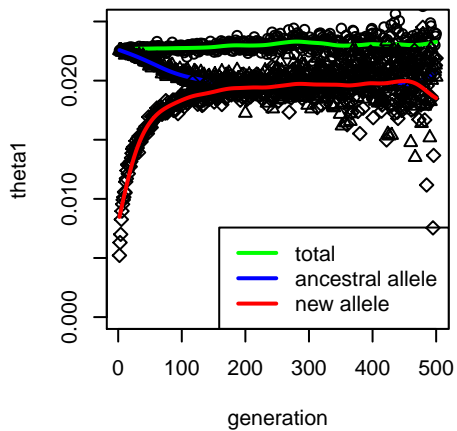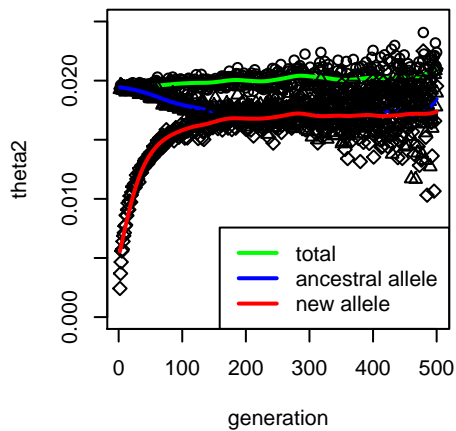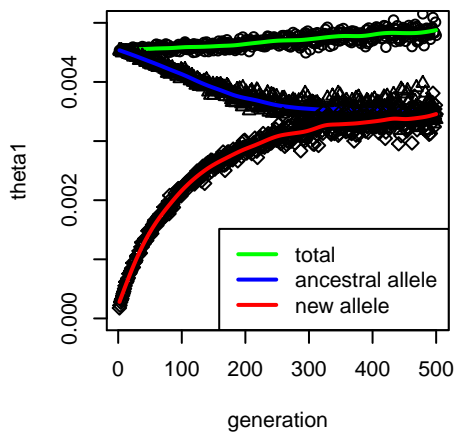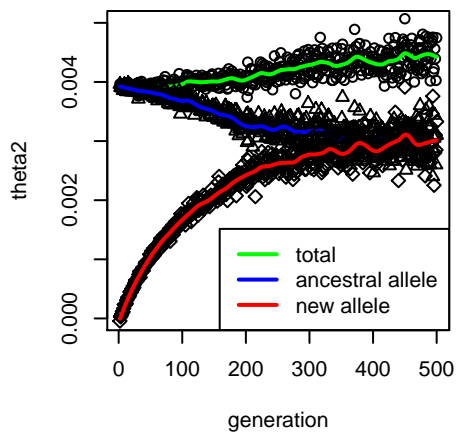

Supplement: S1 Fig — Simulation results of diversity measurements for derived and ancestral alleles: A. N: 100, mutation rate: 0.0001, recombination rate: 0.0001, range: 1000; B. N:50, mutation rate: 0.0001, recombination rate: 0.0001; C. N:100, mutation rate: 0.00001, recombination rate: 0.00001. (PDF) [file pone.0128186.s001.pdf]

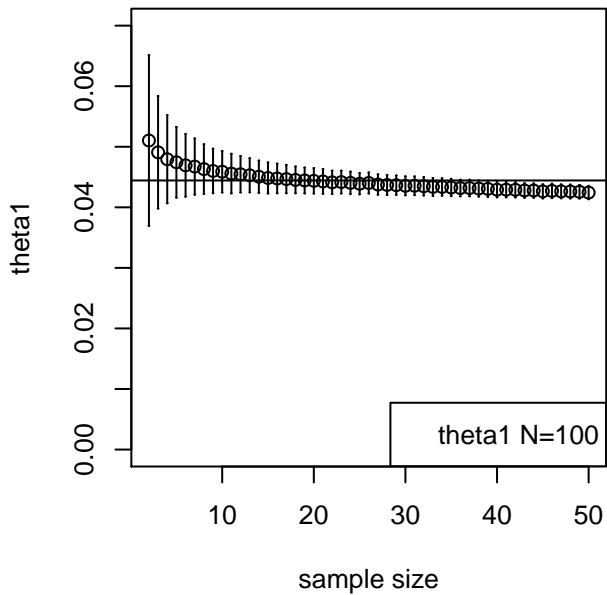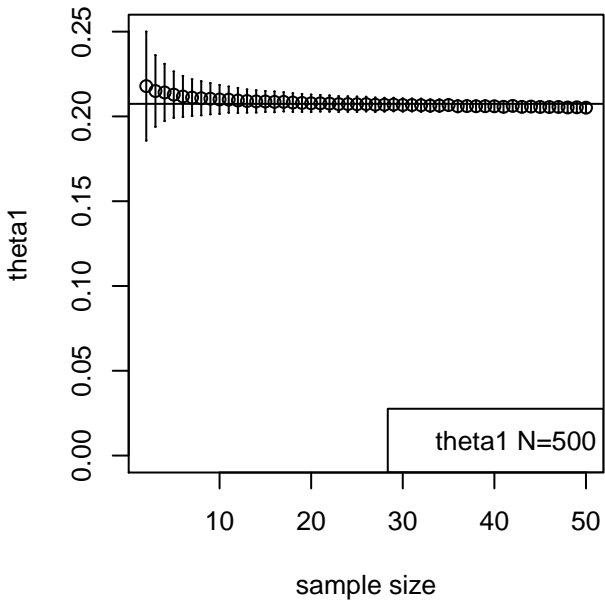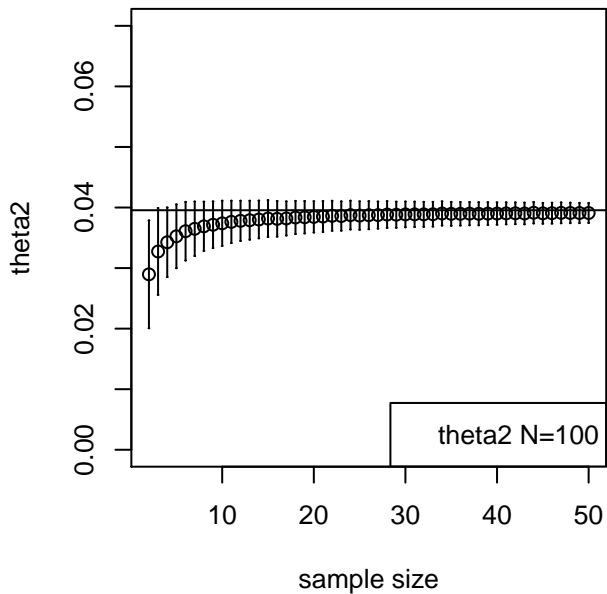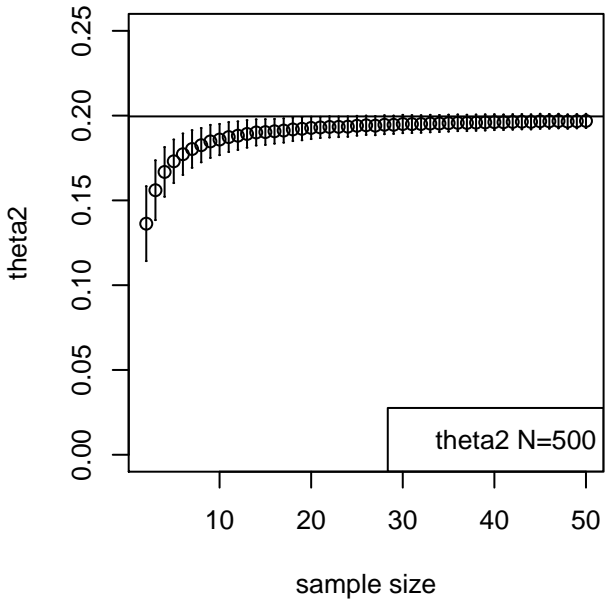

Supplement: S2 Fig — (PDF) [file pone.0128186.s002.pdf]

**A**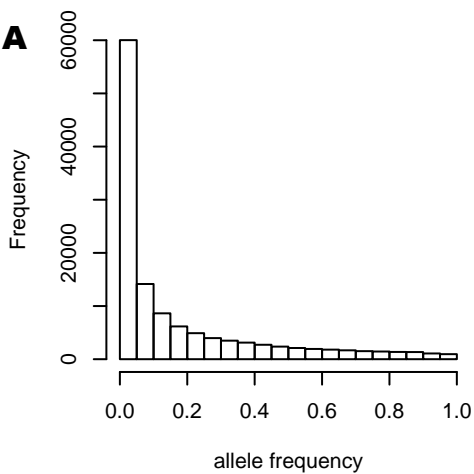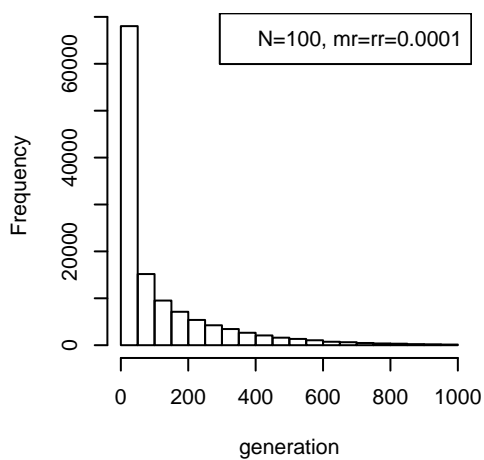**B**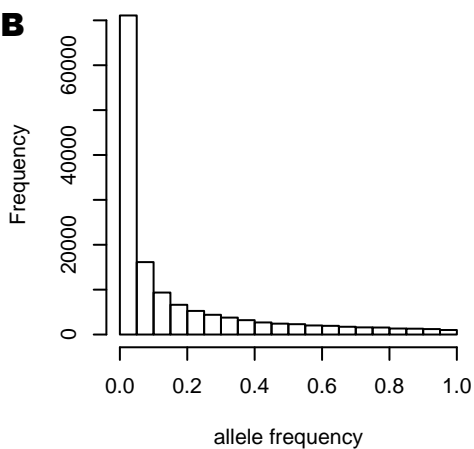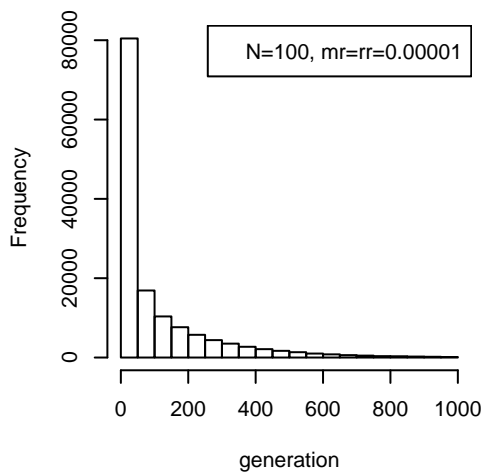**C**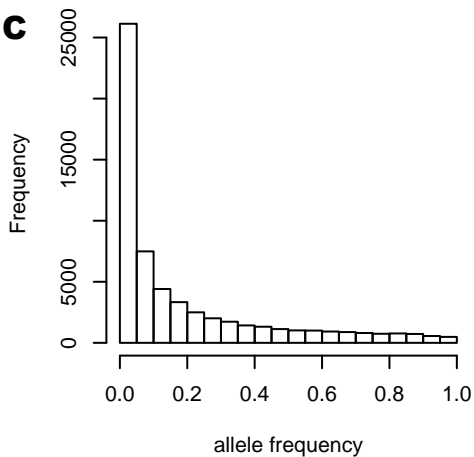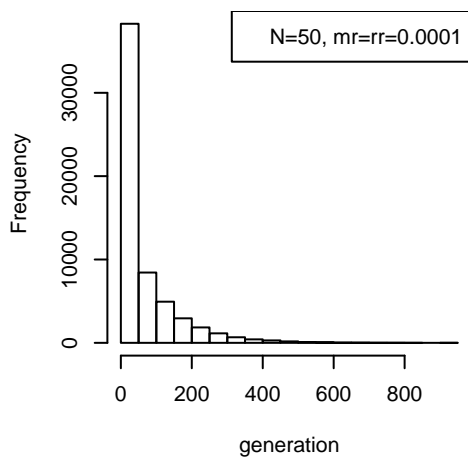

Supplement: S3 Fig — Histograms of derived alleles and distributions of allele ages for various parameters: A. N: 100, mutation rate: 0.0001, recombination rate: 0.0001; B. N:100, mutation rate: 0.00001, recombination rate: 0.00001; C. N:50, mutation rate: 0.0001, recombination rate: 0.0001. (PDF) [file pone.0128186.s003.pdf]

**A. Transitions**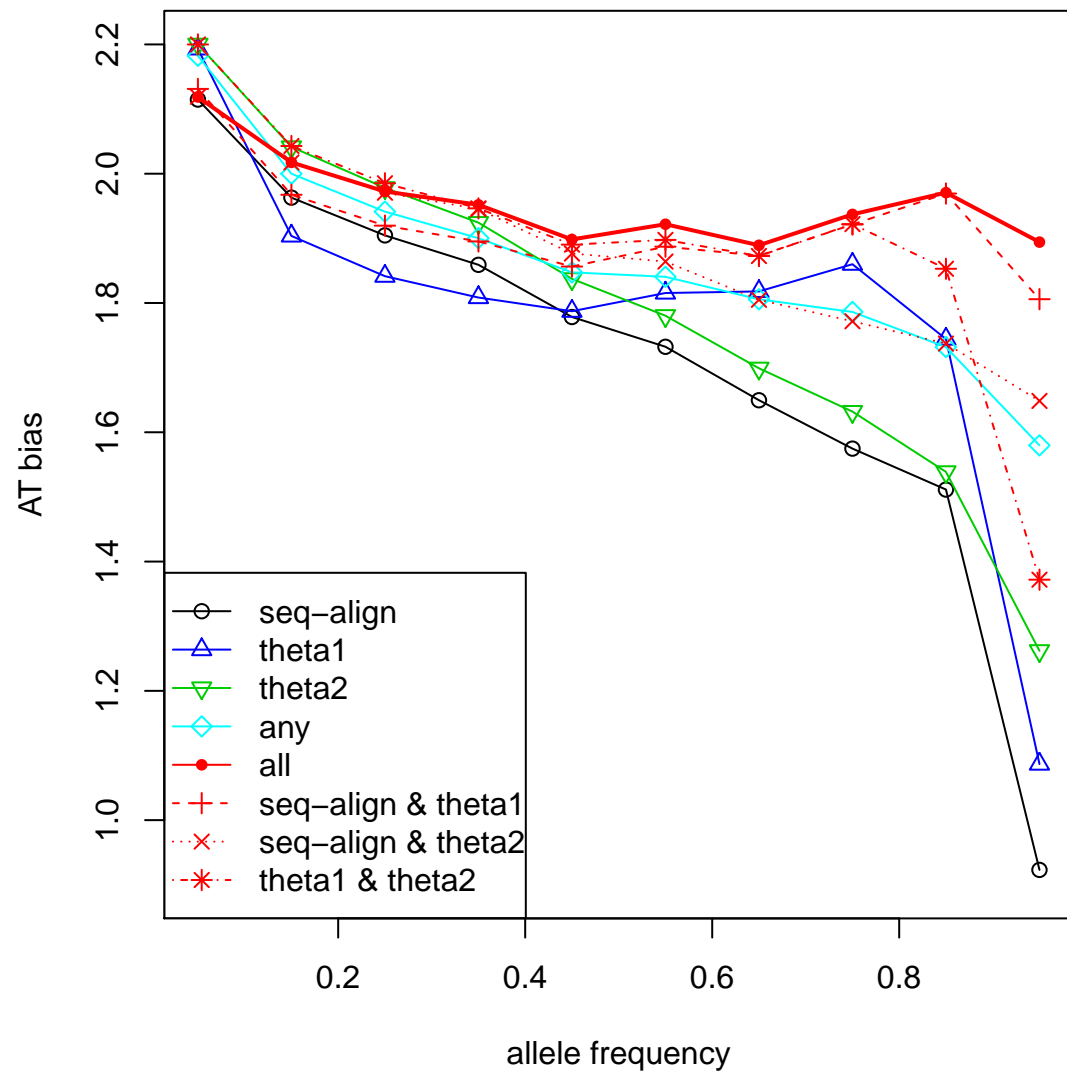**B. Transversions**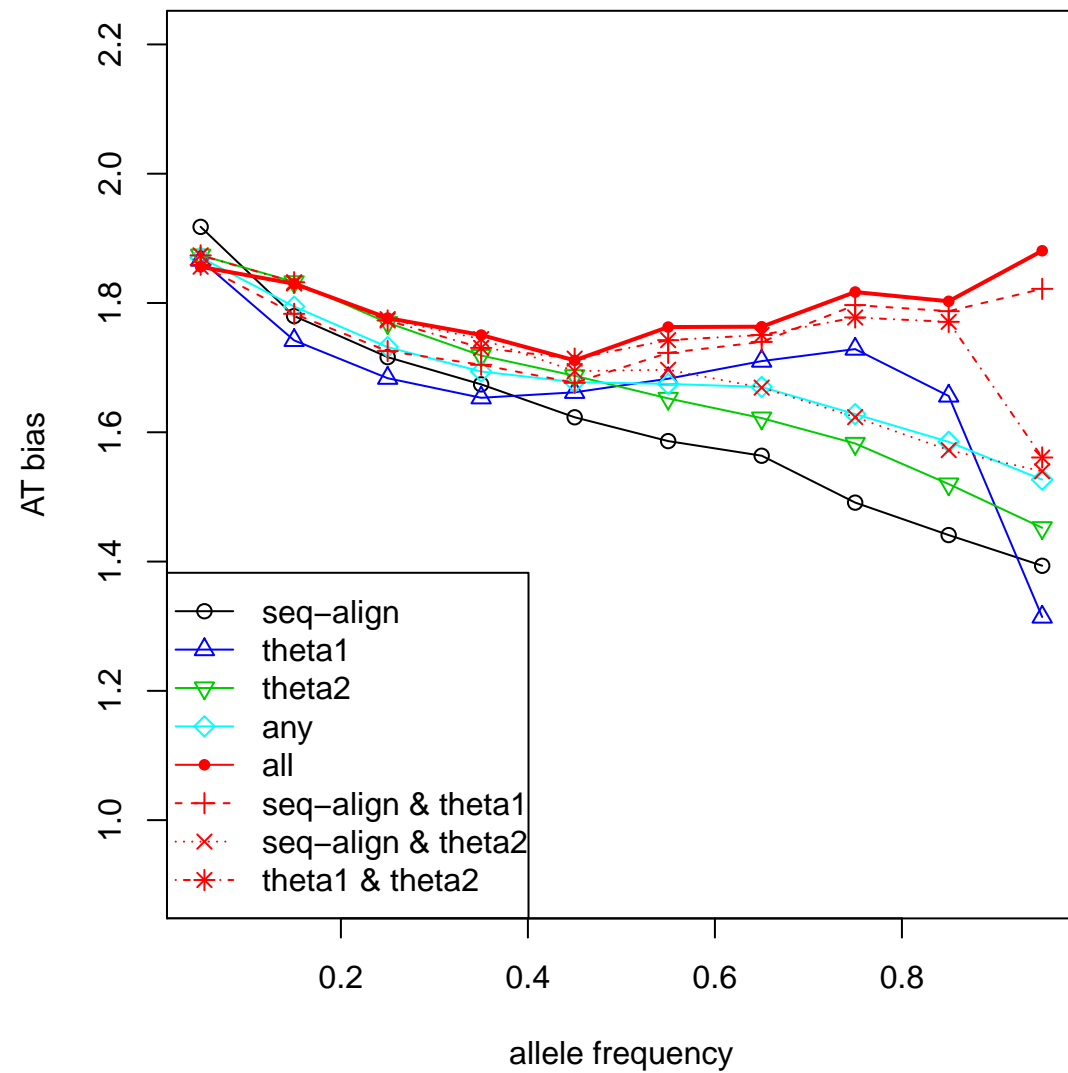

Supplement: S4 Fig — AT bias of nucleotide substitutions depending on derived allele frequencies: A. Transitions; B. Transversions. (PDF) [file pone.0128186.s004.pdf]

**A. EUR exon**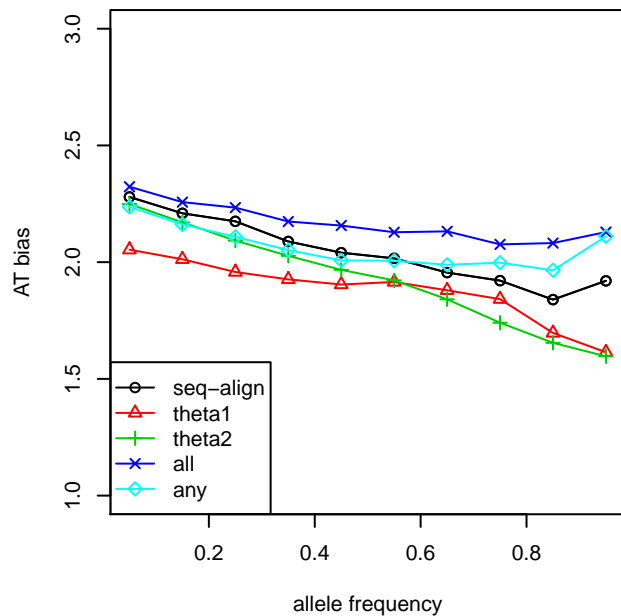**B. EUR gene**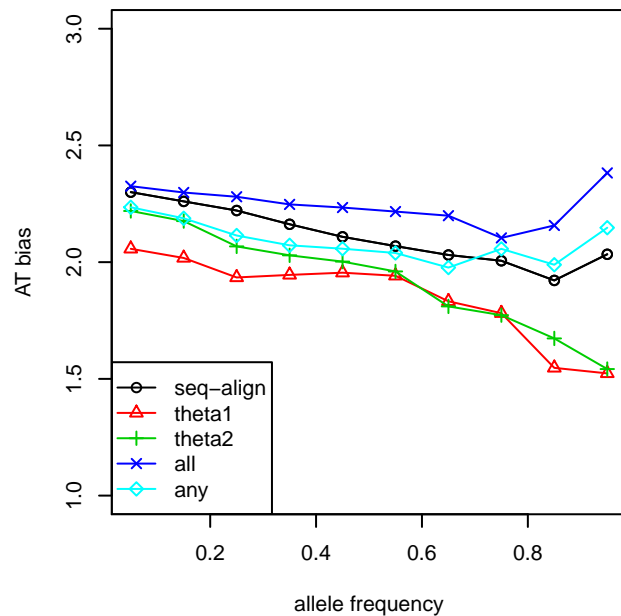**C. EUR repeats**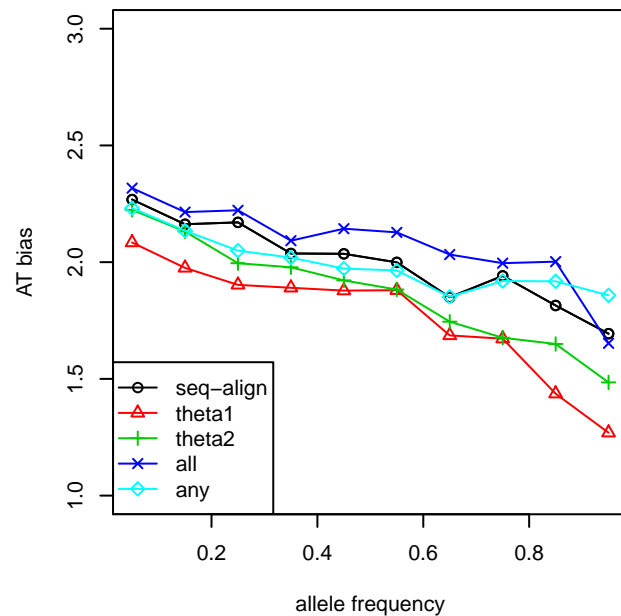**D. EUR trratio**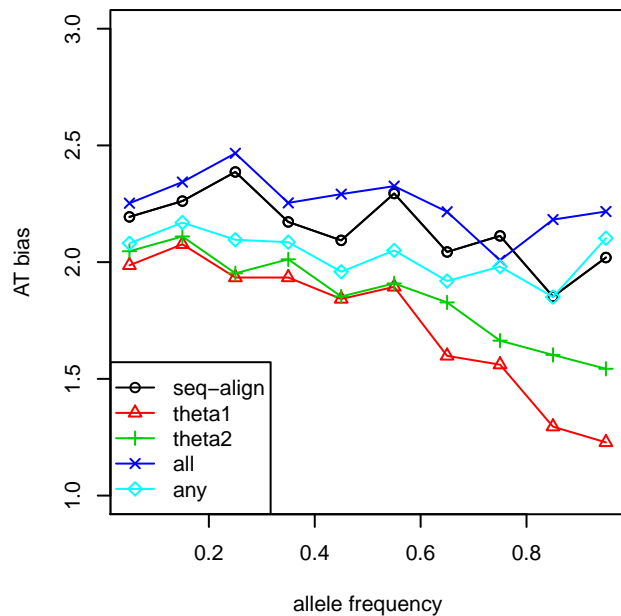**E. EUR total**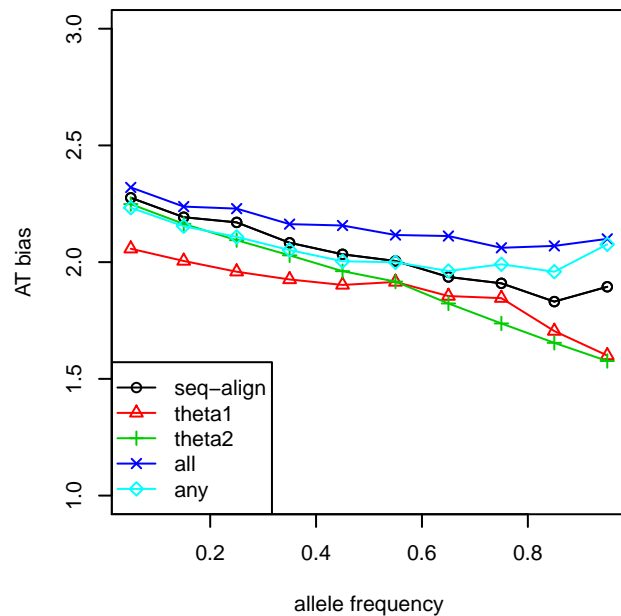**F. YRI total**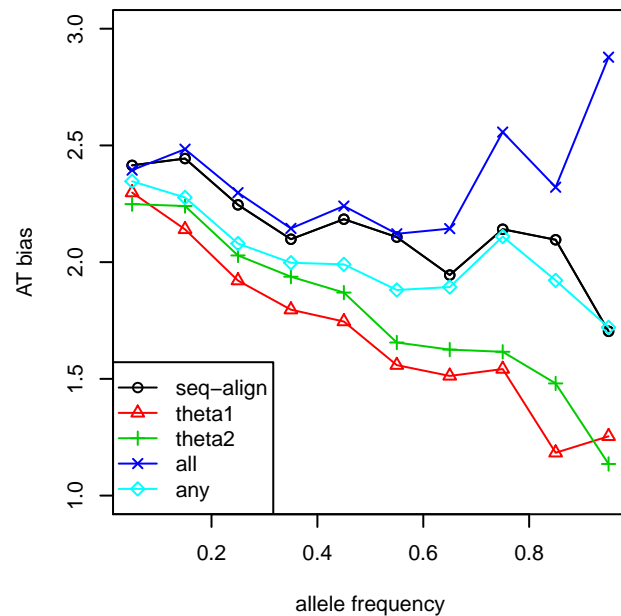

Supplement: S5 Fig — (PDF) [file pone.0128186.s005.pdf]

**A. EUR exon**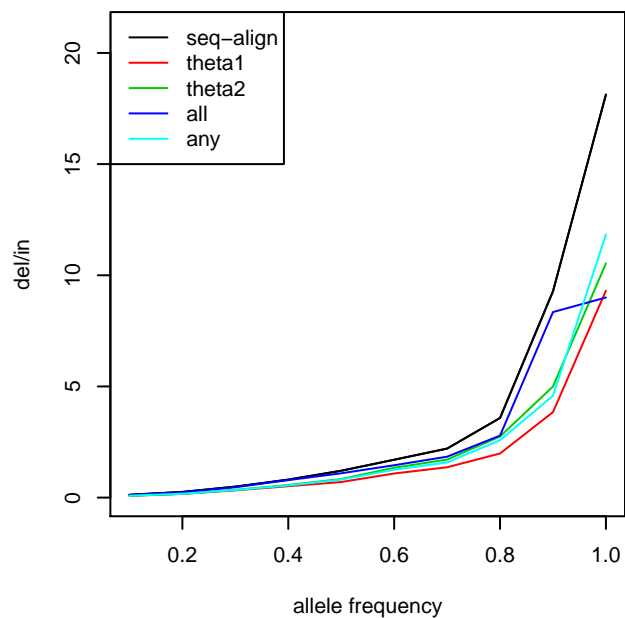**B. EUR gene**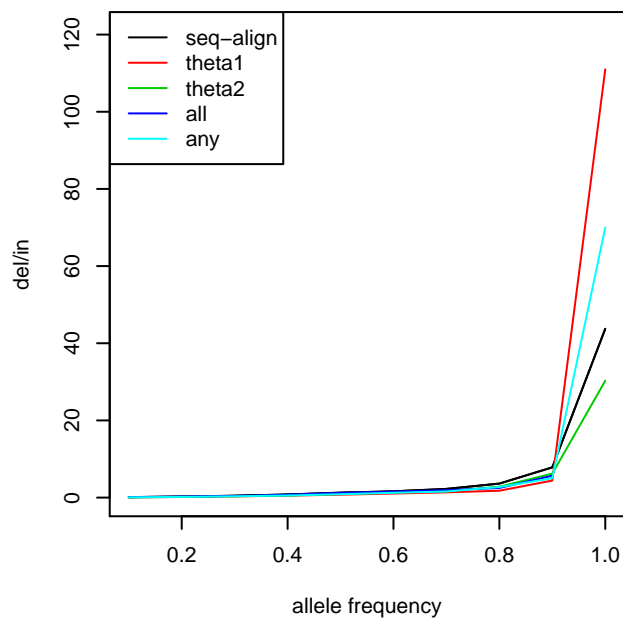**C. EUR repeats**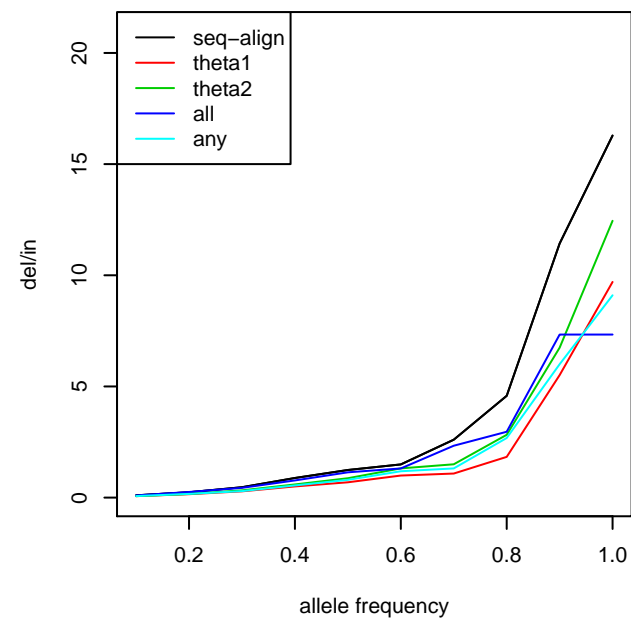**D. EUR ttratio**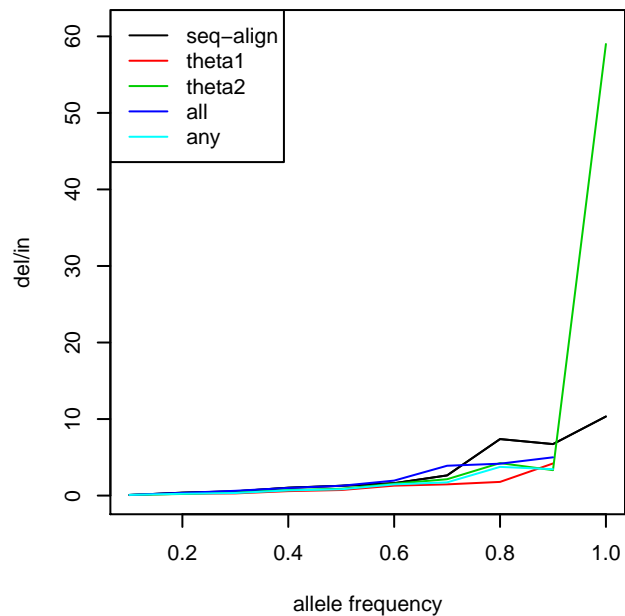**E. EUR total**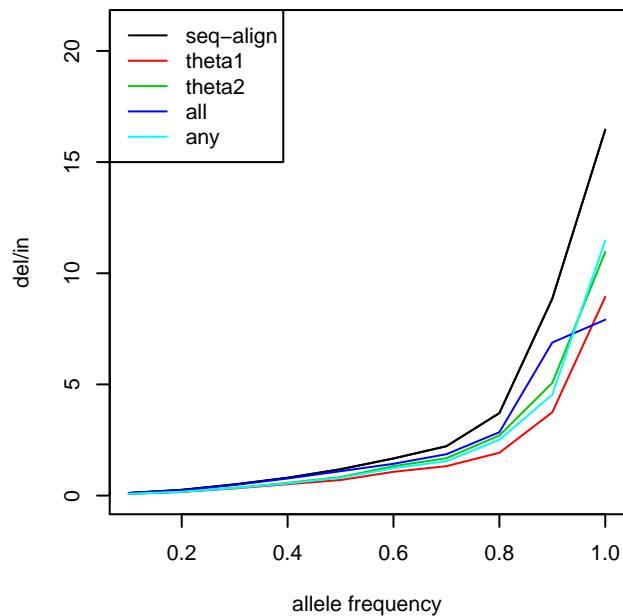**F. YRI total**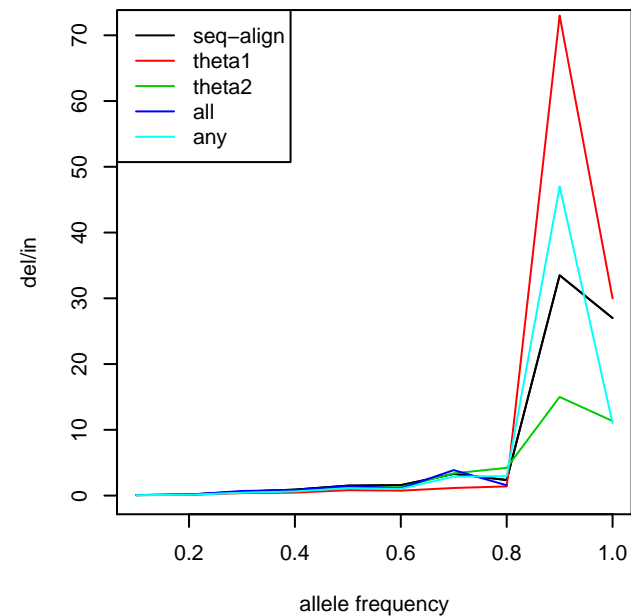

Supplement: S6 Fig — (PDF) [file pone.0128186.s006.pdf]
