# Supplementary material for: Ancestral Alleles in the Human Genome Based on Population Sequencing Data
Source: PLoS One. 2015 May 28;10(5):e0128186. doi: 10.1371/journal.pone.0128186 (PMC4447449; doi:10.1371/journal.pone.0128186)
Supplement: S1 File — Table A. Number of total variants that were examined (QTL: variants, of which positions were not duplicated in QTL data; Analyzed: variants that were successfully merged with data with ancestral allele information; mi: microRNA; exon: exon expression QTL; gene: gene expression QTL; repeats: gene repeats QTL; trratio: transcription ratio QTL; combined total: all QTLs). Table B. Number of nucleotide substitutions for each type of variant when all three methods agreed (DI: deletion and insertion; QTL EUR exon: EUR exon-expresion QTL data; QTL YRI exon: YRI exon-expression QTL data; QTL EUR gene: EUR gene-expression QTL data; QTL EUR repeats: EUR transcribed repeats QTL data; QTL EUR trratio: EUR transcription ratio QTL data; GWAS: GWAS catalog data). (DOC) [file pone.0128186.s007.doc]

|  | **EUR** | | **YRI** | |
| --- | --- | --- | --- | --- |
|  | QTL | Analyzed | QTL | Analyzed |
| **mi** | 3724 | 3530 | 488 | 462 |
| **exon** | 728885 | 690988 | 78157 | 74459 |
| **gene** | 300920 | 285538 | 17328 | 16559 |
| **repeats** | 209946 | 199198 | 20733 | 19791 |
| **trratio** | 65589 | 62404 | 3487 | 3357 |
| **combined total** | 835620 | 792237 | 91338 | 86985 |

Table A. Number of total variants that were examined (QTL: variants, of which positions were not duplicated in QTL data; Analyzed: variants that were successfully merged with data with ancestral allele information; mi: microRNA; exon: exon expression QTL; gene: gene expression QTL; repeats: gene repeats QTL; trratio: transcription ratio QTL; combined total: all QTLs).

| Methods | Direction | AG | CT | AC | GT | AT | CG | DI |
| --- | --- | --- | --- | --- | --- | --- | --- | --- |
| QTL YRI ALL | X → Y | 5066 | 8603 | 1315 | 1673 | 1147 | 1892 | 532 |
|  | X ← Y | 8678 | 5123 | 1693 | 1340 | 1188 | 1911 | 315 |
|  | Total | 13744 | 13726 | 3008 | 3013 | 2335 | 3803 | 847 |
| QTL EUR  ALL | X → Y | 60101 | 95655 | 15736 | 19493 | 13471 | 20425 | 5868 |
|  | X ← Y | 96781 | 60189 | 19692 | 15613 | 13653 | 20719 | 3896 |
|  | Total | 156882 | 155844 | 35428 | 35106 | 27124 | 41144 | 9764 |
| QTL YRI exon | X → Y | 4338 | 7370 | 1094 | 1419 | 965 | 1617 | 447 |
|  | X ← Y | 7383 | 4337 | 1445 | 1123 | 999 | 1633 | 278 |
|  | Total | 11721 | 11707 | 2539 | 2542 | 1964 | 3250 | 725 |
| QTL EUR exon | X → Y | 52527 | 84179 | 13760 | 17029 | 11673 | 17942 | 5140 |
|  | X ← Y | 85100 | 52690 | 17165 | 13644 | 11873 | 18216 | 3420 |
|  | Total | 137627 | 136869 | 30925 | 30673 | 23546 | 36158 | 8560 |
| QTL EUR gene | X → Y | 20112 | 32900 | 5248 | 6708 | 4546 | 7030 | 1958 |
|  | X ← Y | 33342 | 20184 | 6784 | 5319 | 4604 | 7281 | 1423 |
|  | Total | 53454 | 53084 | 12032 | 12027 | 9150 | 14311 | 3381 |
| QTL EUR repeats | X → Y | 13109 | 20353 | 3458 | 4291 | 3005 | 4251 | 1267 |
|  | X ← Y | 20596 | 13070 | 4273 | 3341 | 2996 | 4397 | 903 |
|  | Total | 33705 | 33423 | 7731 | 7632 | 6001 | 8648 | 2170 |
| QTL EUR trratio | X → Y | 3772 | 6242 | 942 | 1193 | 826 | 1203 | 329 |
|  | X ← Y | 6157 | 3681 | 1267 | 878 | 822 | 1291 | 316 |
|  | Total | 9929 | 9923 | 2209 | 2071 | 1648 | 2494 | 645 |
| GWAS | X → Y | 1292 | 1742 | 333 | 375 | 150 | 219 | 2 |
|  | X ← Y | 1651 | 1294 | 380 | 347 | 128 | 214 | 2 |
|  | Total | 2943 | 3036 | 713 | 722 | 278 | 433 | 4 |

Table B. Number of nucleotide substitutions for each type of variant when all three methods agreed (DI: deletion and insertion; QTL EUR exon: EUR exon-expresion QTL data; QTL YRI exon: YRI exon-expression QTL data; QTL EUR gene: EUR gene-expression QTL data; QTL EUR repeats: EUR transcribed repeats QTL data; QTL EUR trratio: EUR transcription ratio QTL data; GWAS: GWAS catalog data).
